# Supplementary material for: Automated neuronal reconstruction with super-multicolour Tetbow labelling and threshold-based clustering of colour hues
Source: Nat Commun. 2024 Jun 25;15:5279. doi: 10.1038/s41467-024-49455-y (PMC11199630; doi:10.1038/s41467-024-49455-y)
Supplement: Supplementary file 1 — Supplementary Information [file 41467_2024_49455_MOESM1_ESM.pdf]

Supplementary Information

Automated neuronal reconstruction with super-multicolour Tetbow labelling and threshold-based clustering of colour hues

Marcus N. Leiwe[1,2,#], Satoshi Fujimoto[1,#], Toshikazu Baba[1,#], Daichi Moriyasu[1], Biswanath Saha[1], Richi Sakaguchi[1], Shigenori Inagaki[1], & Takeshi Imai[1]*

[1] Graduate School of Medical Sciences, Kyushu University, Fukuoka 812-8582, Japan.
[2] Present address: MetaCell LCC, LTD, Cambridge, MA 02142, USA.
[#] These authors contributed equally.
*e-mail: imai.takeshi.457@m.kyushu-u.ac.jp

Supplementary Fig. 1 | Linearity correction

Supplementary Fig. 2 | Linear unmixing separates 7 XFPs.

Supplementary Fig. 3 | Another example of linear unmixing.

Supplementary Fig. 4 | Distinguishing between single-coloured vs. dual-coloured cells.

Supplementary Fig. 5 | Additional modelling data.

Supplementary Fig. 6 | Modelling colours in neurite fragments with noise.

Supplementary Fig. 7 | Quality control for neurite fragment quantifications.

Supplementary Fig. 8 | Optimization of the threshold distance, *Th(d)*.

Supplementary Fig. 9 | Investigation into a neuron represented by multiple clusters.

Supplementary Fig. 10 | Optimization for axonal reconstruction.

Supplementary Fig. 11 | Evaluation of mitral/tufted cell axons identified with QDyeFinder.

Supplementary Fig. 12 | Comparison with existing auto-tracing software.

Supplementary Fig. 13 | Comparison of clustering algorithms in QDyeFinder.

Supplementary Fig. 14 | Comparison between DBSCAN and dCrawler.

Supplementary Table 1 | Comparison between different strategies for neurite reconstruction.

Supplementary Note 1 | Conversion of Imaris files to SWC format, related to Fig. 3.

Supplementary Note 2 | dCrawler description, related to Fig. 4.

Supplementary Data 1 | Evaluation of colour consistency in dendrites, related to Fig. 5b.

Supplementary Data 2 | Comparison of dCrawler clusters vs. ground truth for dendrites, related to Fig. 5f.

Supplementary Data 3 | Evaluation of colour consistency in axons, related to Supplementary Fig. 7.

Supplementary Data 4 | Comparison of dCrawler clusters vs. ground truth for axons, related to Supplementary Fig. 7.

Supplementary Data 5 | Parameters used for linear unmixing in this study, related to Methods.

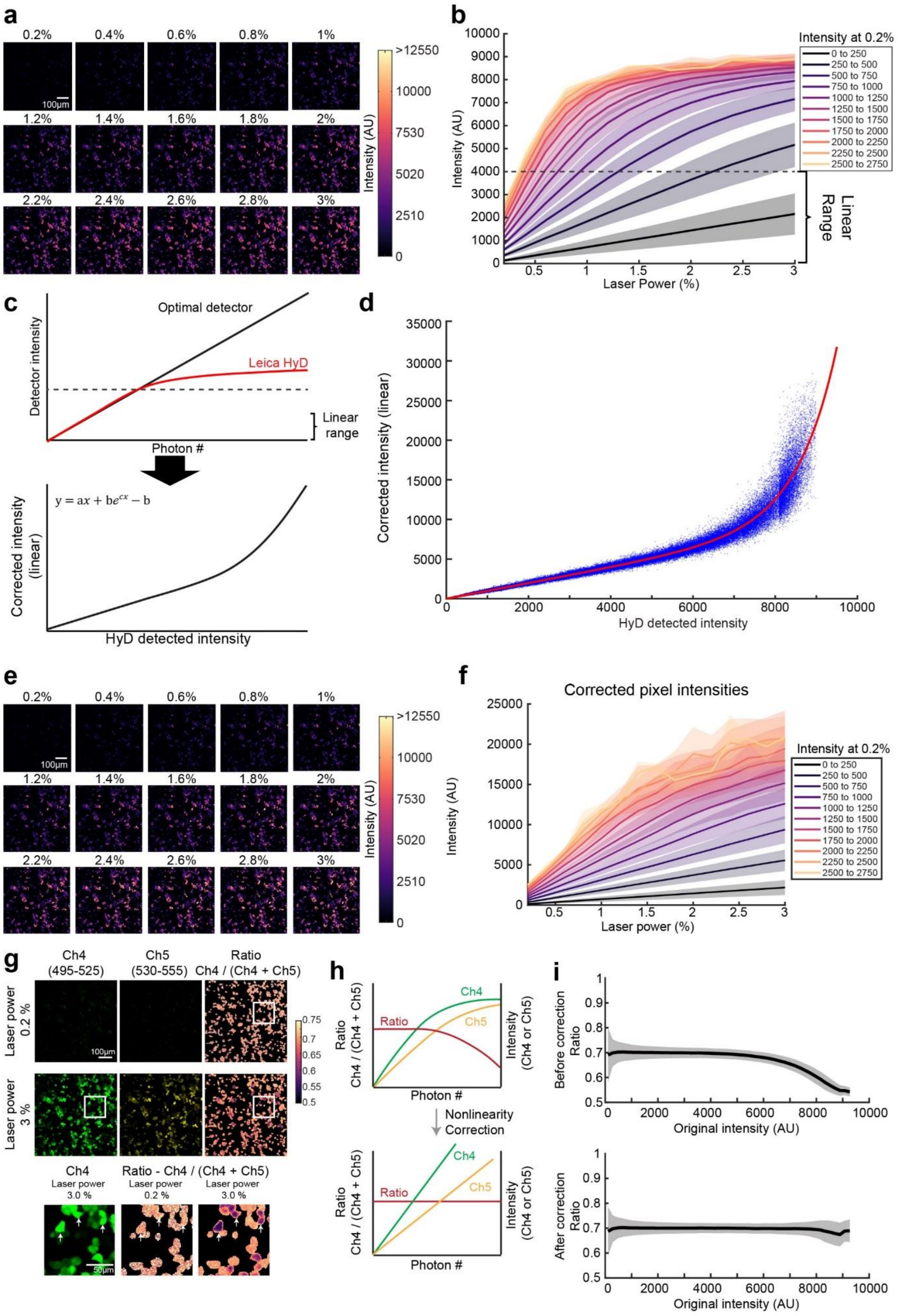

a
0.2% 0.4% 0.6% 0.8% 1%
1.2% 1.4% 1.6% 1.8% 2%
2.2% 2.4% 2.6% 2.8% 3%
100μm
Intensity (AU)
>12550 10000 7530 5020 2510 0
b
Intensity at 0.2%
0 to 250
250 to 500
500 to 750
750 to 1000
1000 to 1250
1250 to 1500
1500 to 1750
1750 to 2000
2000 to 2250
2250 to 2500
2500 to 2750
Intensity (AU)
Laser Power (%)
Linear Range
c
Optimal detector
Leica HyD
Detector intensity
Photon #
Linear range
$y = ax + be^{cx} - b$
Corrected intensity (linear)
HyD detected intensity
d
Corrected intensity (linear)
HyD detected intensity
e
0.2% 0.4% 0.6% 0.8% 1%
1.2% 1.4% 1.6% 1.8% 2%
2.2% 2.4% 2.6% 2.8% 3%
100μm
Intensity (AU)
f
Corrected pixel intensities
Intensity at 0.2%
Intensity (AU)
Laser power (%)
g
Ch4 (495-525)
Ch5 (530-555)
Ratio Ch4 / (Ch4 + Ch5)
Laser power 0.2 %
Laser power 3 %
100μm
Ch4
Ratio - Ch4 / (Ch4 + Ch5)
Laser power 3.0 %
Laser power 0.2 %
Laser power 3.0 %
50μm
h
Ratio
Ch4
Ch5
Ratio Ch4 / (Ch4 + Ch5)
Intensity (Ch4 or Ch5)
Photon #
Nonlinearity Correction
i
Before correction Ratio
Original intensity (AU)
After correction Ratio
Original intensity (AU)

Supplementary Fig. 1 | Linearity correction

a, HEK293T cells transfected with mNeonGreen were cleared with SeeDB2G and imaged at laser powers from 0.2% to 3% to examine the saturation curve of the Leica HyD detectors. Images were acquired under “standard mode” at 16-bit. Laser powers were changed linearly with AOTF.

b, Intensities of pixels as the laser power increases, after the intensity goes beyond 4000 pixels begin to saturate. For the plot, pixels were grouped according to their intensity values at the lowest laser power with the mean (line) ± standard deviation (shaded area) displayed for each group.

c, Top panel, cartoon plot highlighting the difference between an ideal linear detector (black line) compared to the Leica HyD detector which begins to saturate (red line). Bottom panel, the values of our ideal detector can be predicted by extrapolating from the linear range of the HyD detector. An equation can then be fitted and used to convert values from the HyD detector to the linear values.

d, Pixels from the images in **a** were used to determine the equation used to convert intensities. Intensities above 8,000 are still noisy and a poor fit.

e, Corrected images from **a**, showing the improved linearity of the image, especially in the bright range.

f, Corrected intensities of pixels as the laser power increases. Pixels were binned in the same manner as in **b**.

g, Evaluation of non-linearity correction on the ratio of two different fluorescent proteins (Ch4 for mNeonGreen, and Ch5 for YPet). Raw intensity values before unmixing were used to calculate the ratio, Ch4 / (Ch4 + Ch5). Top panel shows the images and ratios at a low laser power (0.2%). Middle panel shows the images and ratios at a high laser power (3%). Bottom panels show the zoomed in areas of the ratiometric image (white box). Note that ratiometric images have an applied mask to remove background signal. White arrows indicate examples where the intensity ratios change with differences in laser power.

h, Schematic cartoon predicting the improvement in the consistency of the ratio values, Ch4 / (Ch4 + Ch5), at all levels of brightness.

i, Changes in ratio, Ch4 / (Ch4 + Ch5), with laser power before and after non-linear correction. Line represents the mean, and the shaded area represents ±1 standard deviation.

AU, arbitrary unit.

Source data are provided as a Source Data file.

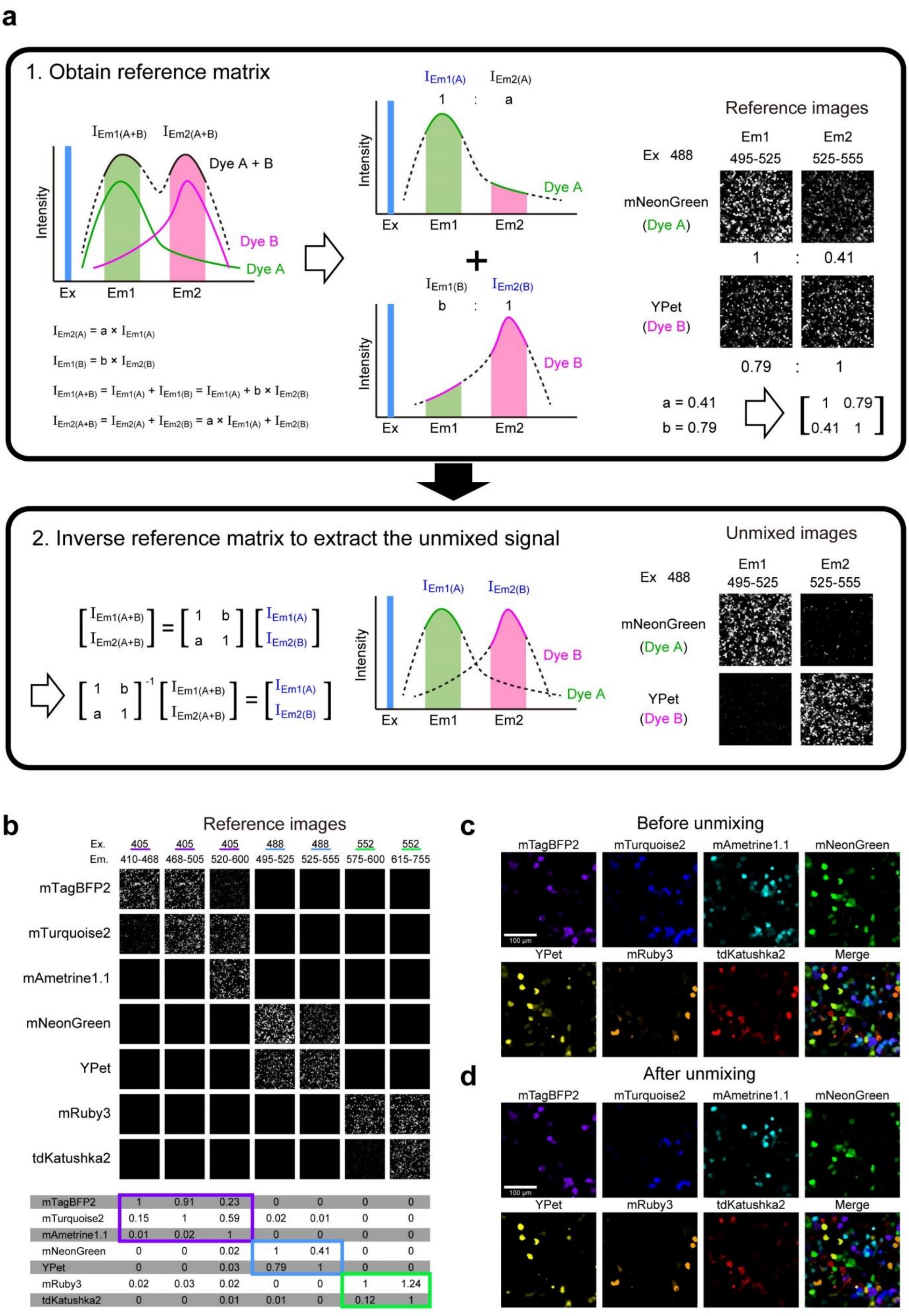

mTagBFP2	1	0.91	0.23	0	0	0	0
mTurquoise2	0.15	1	0.59	0.02	0.01	0	0
mAmetrine1.1	0.01	0.02	1	0	0	0	0
mNeonGreen	0	0	0.02	1	0.41	0	0
YPet	0	0	0.03	0.79	1	0	0
mRuby3	0.02	0.03	0.02	0	0	1	1.24
tdKatushka2	0	0	0.01	0.01	0	0.12	1

Supplementary Fig. 2 | Linear unmixing separates 7 XFPs.

a, Cartoon to explain the process of linear unmixing. Box 1 left shows the problem when two dyes have spectral overlap. The intensity value recorded by the channel is the sum of intensities of both dye A and dye B. We can then record the reference intensities for both channels recording them as a ratio for each dye (1:a, and b:1 respectively), which can then be expressed as a matrix. The recorded intensity can be expressed as the matrix multiplication of our reference matrix and the intensity signal generated by each dye separately. This means we can rearrange the equation to calculate the intensity signal generated by each dye separately.

b, To generate our reference matrices, HEK293T cells transfected with a single XFP were imaged. There reference values were used to unmix the images in Fig. 1c.

c-d, A mixture of HEK293T cells, each expressing just one type out of 7 XFPs. Images before **(c)** and after **(d)** linear unmixing. We were able to remove spectral overlap so that no cells were double labelled.

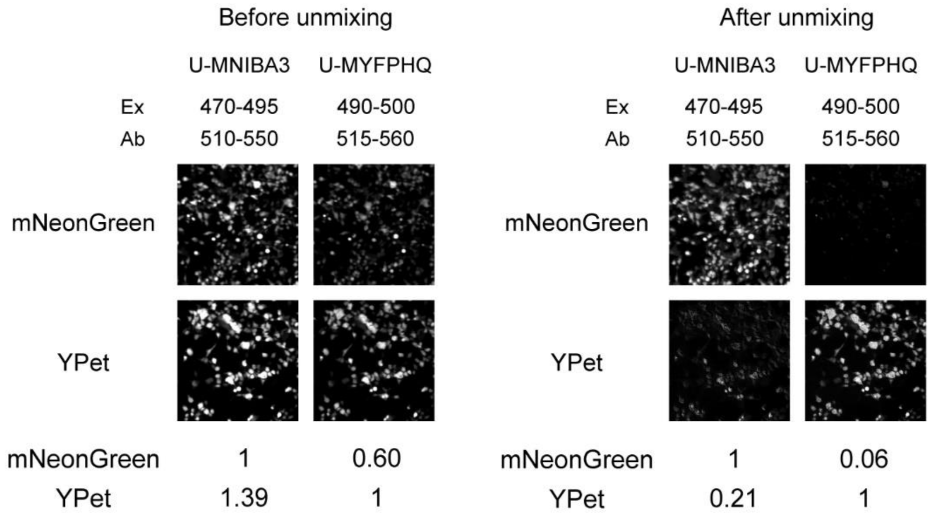

Supplementary Fig. 3 | Another example of linear unmixing.

In this study, we used spectral dispersion prism to separate fluorescence signals with Leica confocal microscope, SP8. However, linear unmixing is applicable to images taken with conventional fluorescence microscopes equipped with conventional filter/mirror units. Therefore, we can use our QDyeFinde pipeline for any kinds of conventional confocal microscopy. Here, HEK293T cells expressing mNG or YPet are excited and emitted through two different fluorescent mirror units (U-MNIBA3 or U-MYFPHQ, Olympus) using Olympus epifluorescence microscope, MVX-10. Excitation (Ex) and absorption (Ab) ranges of each mirror units are indicated. The images before unmixing (left) and after unmixing (right) are shown.

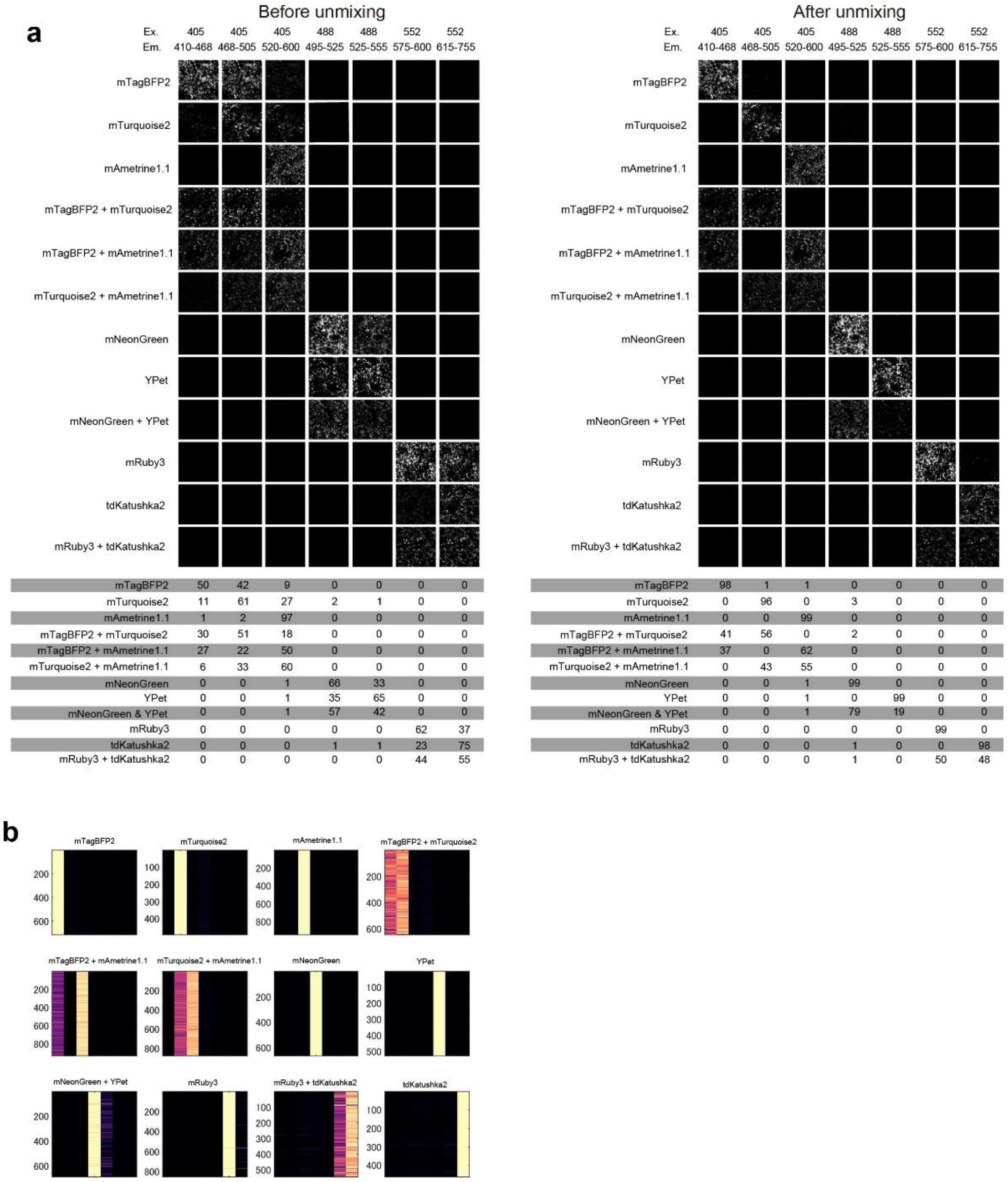

Before unmixing

Ex. / Em.	405 / 410-468	405 / 468-505	405 / 520-600	488 / 495-525	488 / 525-555	552 / 575-600	552 / 615-755
mTagBFP2	50	42	9	0	0	0	0
mTurquoise2	11	61	27	2	1	0	0
mAmetrine1.1	1	2	97	0	0	0	0
mTagBFP2 + mTurquoise2	30	51	18	0	0	0	0
mTagBFP2 + mAmetrine1.1	27	22	50	0	0	0	0
mTurquoise2 + mAmetrine1.1	6	33	60	0	0	0	0
mNeonGreen	0	0	1	66	33	0	0
YPet	0	0	1	35	65	0	0
mNeonGreen & YPet	0	0	1	57	42	0	0
mRuby3	0	0	0	0	0	62	37
tdKatushka2	0	0	0	1	1	23	75
mRuby3 + tdKatushka2	0	0	0	0	0	44	55

After unmixing

Ex. / Em.	405 / 410-468	405 / 468-505	405 / 520-600	488 / 495-525	488 / 525-555	552 / 575-600	552 / 615-755
mTagBFP2	98	1	1	0	0	0	0
mTurquoise2	0	96	0	3	0	0	0
mAmetrine1.1	0	0	99	0	0	0	0
mTagBFP2 + mTurquoise2	41	56	0	2	0	0	0
mTagBFP2 + mAmetrine1.1	37	0	62	0	0	0	0
mTurquoise2 + mAmetrine1.1	0	43	55	0	0	0	0
mNeonGreen	0	0	1	99	0	0	0
YPet	0	0	1	0	99	0	0
mNeonGreen & YPet	0	0	1	79	19	0	0
mRuby3	0	0	0	0	0	99	0
tdKatushka2	0	0	0	1	0	0	98
mRuby3 + tdKatushka2	0	0	0	1	0	50	48

Supplementary Fig. 4 | Distinguishing between single-coloured vs. dual-coloured cells.

a, We examined whether we can distinguish between single-coloured vs. dual-coloured cells after the linear unmixing. HEK293T cells expressing one or two types of 7 XFPs before (left panels) and after (right panels) linear unmixing. When expressing two XFPs, cells were transfected using a mixture of the plasmid: CAG-tTA2 (0.35 μg/dish) and both pAAV-TRE-XFP1 and pAAV-TRE-XFP2 (0.45 μg/dish each). Reference data for the linear unmixing was acquired from another set of images. Percentages of normalized intensities are shown below.

b, The vector normalized color vector of each barcode. Columns indicate individual cells that were separated using Cellpose.

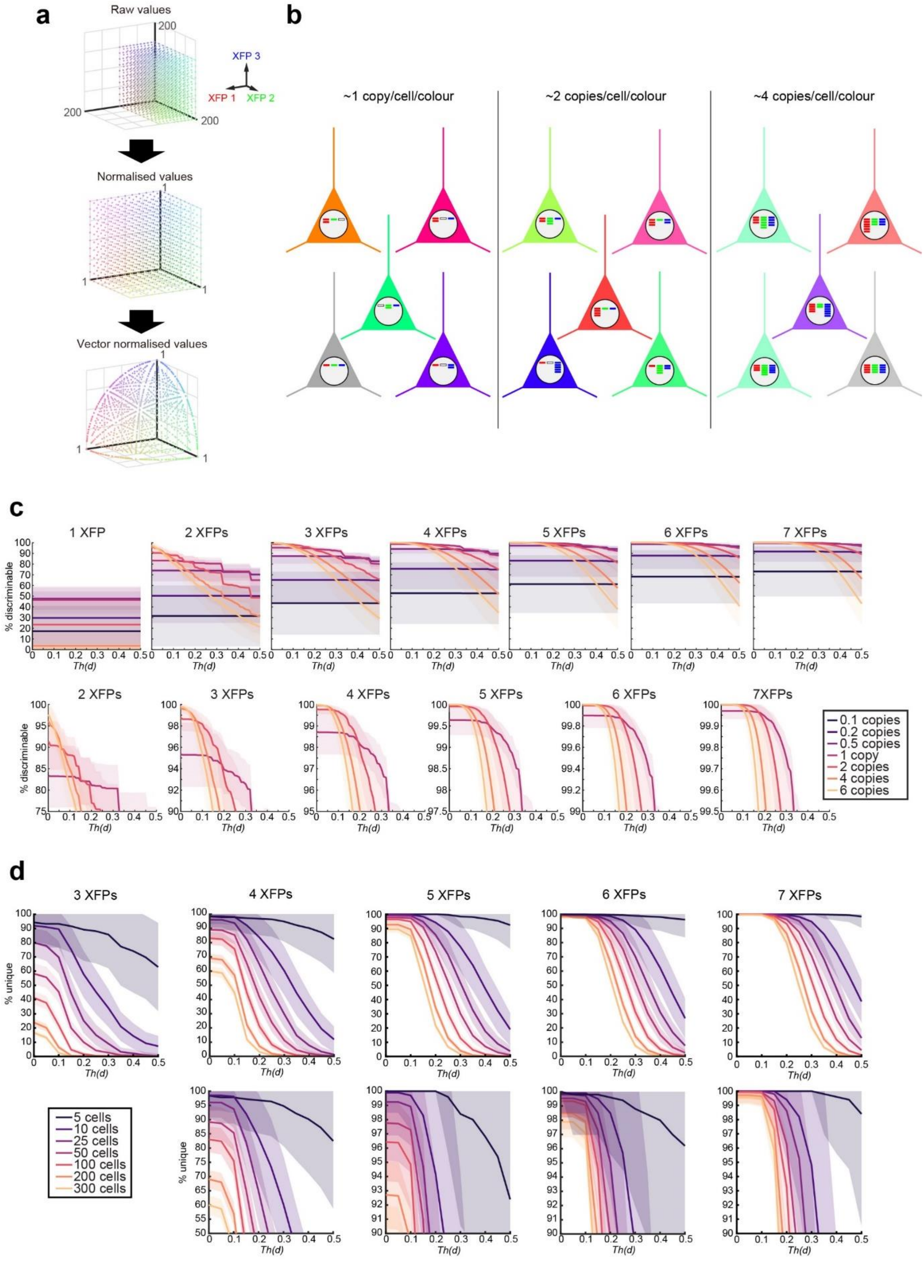

Supplementary Fig. 5 | Additional modelling data.

a, Cartoon showing the conversion of 3-channel fluorescence signals into vector normalised colour vectors. The same scheme was used for N-channel images.

b, Copy number of XFP genes follow Poisson distributions in Tetbow method. Cartoon shows the effect of plasmid/AAV concentrations for 3 XFPs.

c, More detailed investigation of the percent discriminable for 1-7 XFPs at 0.1, 0.2, 0.5, 1, 2, 4, and 6 copies per colour per cell. Top row displays the overall patterns at a *Th(d)* from 0 to 0.5. Bottom row displays a zoomed in version showing the differences between the copy numbers at tighter range. Data are mean ± SD (n = 10,000 each).

d, More detailed investigation of the percent of cells unique at 3-7 XFPs when 5, 10, 25, 50, 100, 200, and 300 cells are labelled at 2 copies per cell per colour. Top panels display a broad organisation of pattern for the different number of cells labelled at each number of XFPs. Bottom row contains a zoomed in view of the data in the top row.

Source data are provided as a Source Data file.

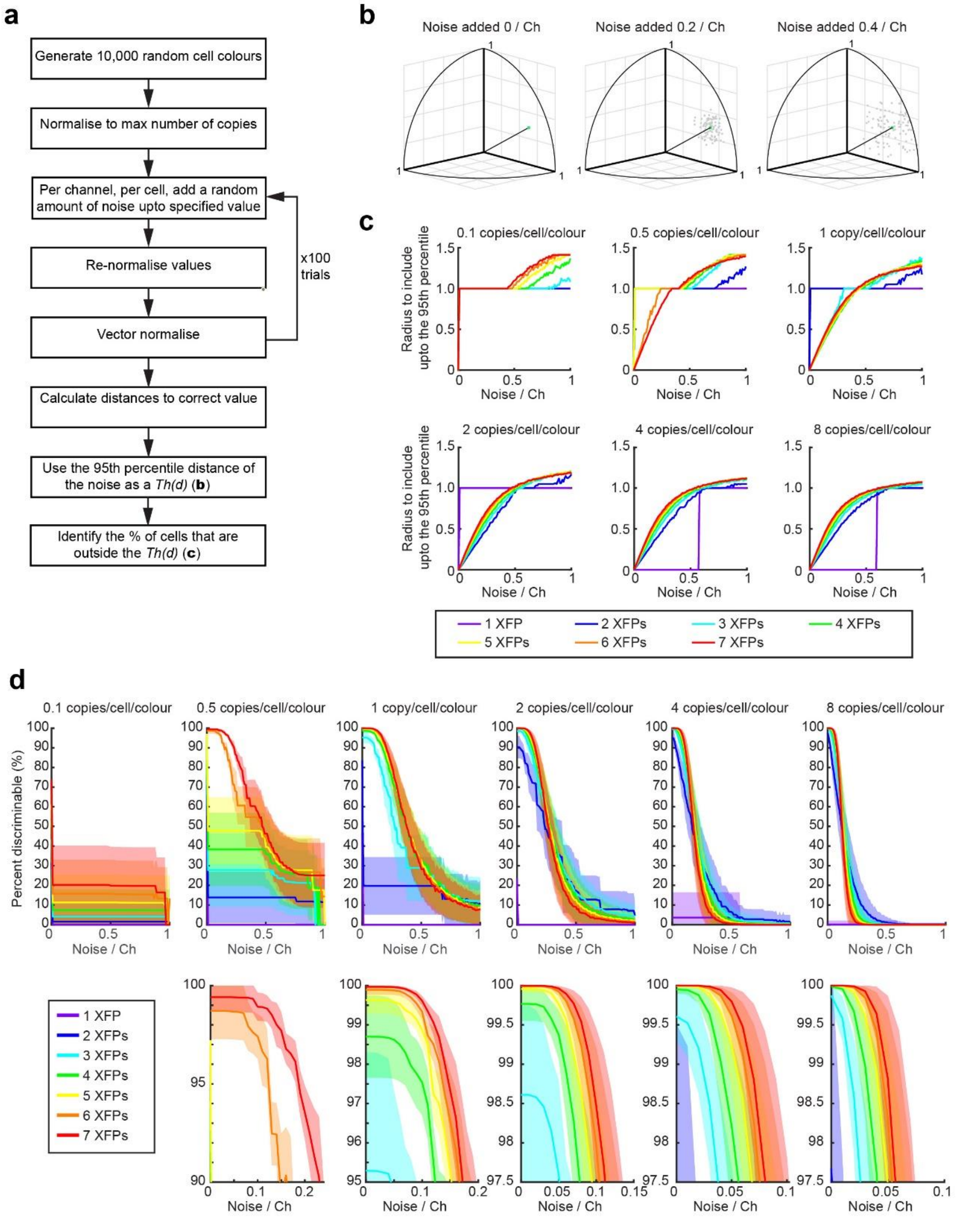

Supplementary Fig. 6 | Modelling colours in neurite fragments with noise.

In-silico modelling of the neurite signals with noise introduced by increasing the number of colour dimensions at different copy numbers per cell.

a, Workflow of the simulations, as with previous modelling 10,000 "cells" with a vector normalised Poisson colour distribution were generated. For each channel per cell a random amount of noise was added up to a specified amount (**b**), and the new colour value for "neurites" was calculated 100 times. The noise follows the normal distribution and the *x*-axis

in **c** and **d** (noise) represent the SD of the noise per channel. The 95th percentile of the distances to the original colour was then used as the threshold distance, *Th(d)* (**c**), to calculate the percent discriminable of the original 10,000 cells (**d**).

b, Diagram displaying the effect of introducing various amounts of noise per channel. As the noise increases so does the diameter of points.

c, Changes in the threshold value calculated varies across the different copy numbers. As the number of dimensions increases so do the corresponding radii (measure of noise).

d, The percent of cells discriminable (*y*-axis) as increasing amounts of noise are introduced (*x*-axis) at different dimensions (coloured lines) and different copy numbers (subplots). Despite the increased amounts of noise in higher dimensions, the percentage of cells discriminable is still higher.

Source data are provided as a Source Data file.

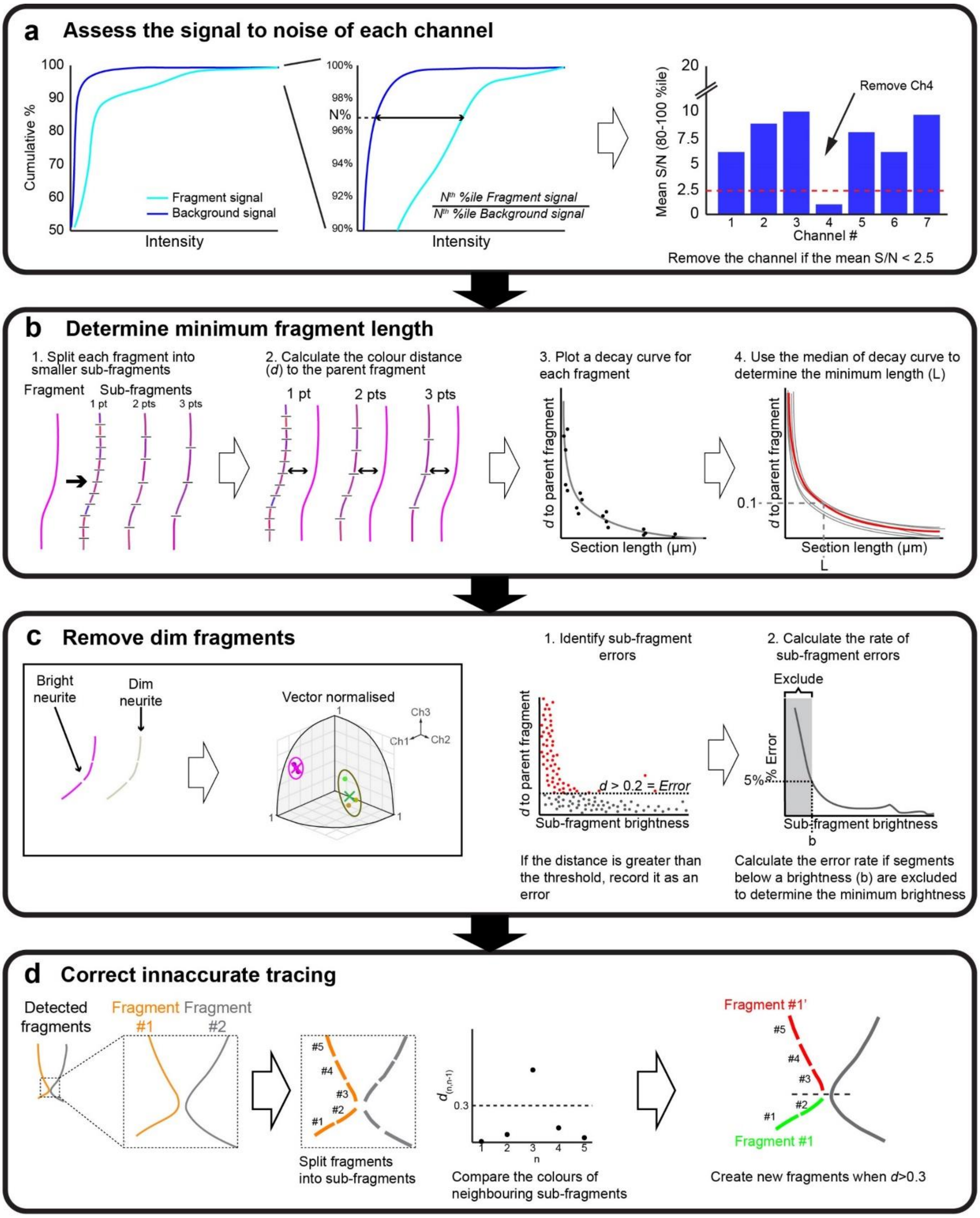

Supplementary Fig. 7 | Quality control for neurite fragment quantifications.

a, Firstly, the overall quality of each channel was assessed by comparing the signal to noise ratio of the voxels labelled in the neurite fragments (cyan) and the background/non-labelled voxels (blue). The mean signal to noise ratio was calculated for each percentile between 80-100 (infinite values excluded). If the mean signal to noise ratio was above 2.5, the channel was retained.

b, Determining the minimum fragment length. Shorter fragments often produce inaccurate colour vectors as they do not have sufficient voxels to produce a stable mean value. To calculate the minimum length of a fragment that produces a stable colour vector, each neurite fragment was split into sub-fragments of varying lengths (leftmost cartoon). The colour distance (*d*) between each sub-fragment and the parent fragment was measured (second left plot), for each fragment a decay curve is fitted (step 3). Finally, the decay curves for each fragment are used to calculate a median decay curve. The minimum length is then calculated at where the curve crosses $d = 0.1$.

c, Dim fragments can also result in inaccurate colour vectors (left panel). Brightness can be measured for a fragment before vector normalisation. To establish the minimum brightness necessary, the fragment was split into sub-fragments each at the minimum distance calculated in **b**. Then the brightness was calculated before vector normalisation. After vector normalization to obtain the colour vectors, distance to the parent fragment (*d*) was measured. If the colour distance was above the specified threshold, it was marked as an error (middle panel). Finally, to calculate the minimum brightness necessary, the error percentage was calculated if sub-fragments less than a particular brightness were excluded. From this line the first brightness value below an error threshold (5%) was selected to be the minimum brightness (b) of a threshold.

d, Another possible error that can emerge from Neurolucida 360 fragment detection. When neurites cross each other, the tracer (automated or manual) can make a wrong decision (left). To correct for this, the fragments were split into small sub-fragments as in **b** and the colour distance (*d*) between neighbouring sub-fragments was measured (middle). If *d* was greater than the specified value (0.3), the fragment was split into two at that point (right).

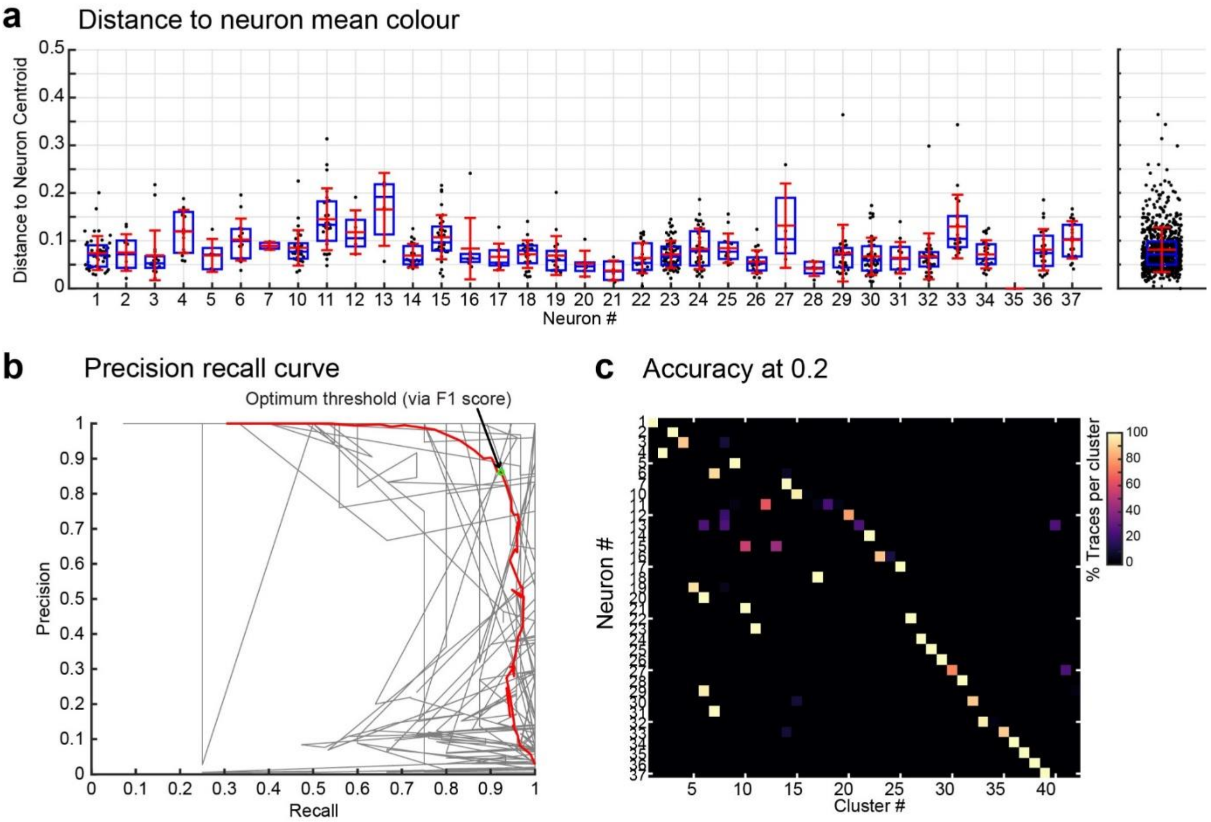

Supplementary Fig. 8 | Optimization of the threshold distance, *Th(d)*.

a, The initial variation of colour vectors per cell. Distance to the centroid is shown. Each fragment is represented by a grey point, mean (red horizontal bar), median (blue horizontal bar), ± 1 standard deviation (red whiskers), and the interquartile range (blue box). Panel on the right shows all the points together.

b, Precision recall curves for each manually traced neuron (grey lines) and the median (red line), with the position of the optimal threshold indicated (green circle). Each point in each curve represents a *Th(d)* ranging from 0.05 to 1. Precision is defined as the number of true positives divided by the sum of the true positives and false positives. Recall is defined as the number of true positives divided by the sum of the true positives and the false negatives.

c, Matrix that displays the percentage contribution of fragments belonging to each cluster (*x*-axis) to the 37 manually traced neurons (*y*-axis). 100% represents the situation where a single cluster includes all the fragments associated with the neuron.

Source data are provided as a Source Data file.

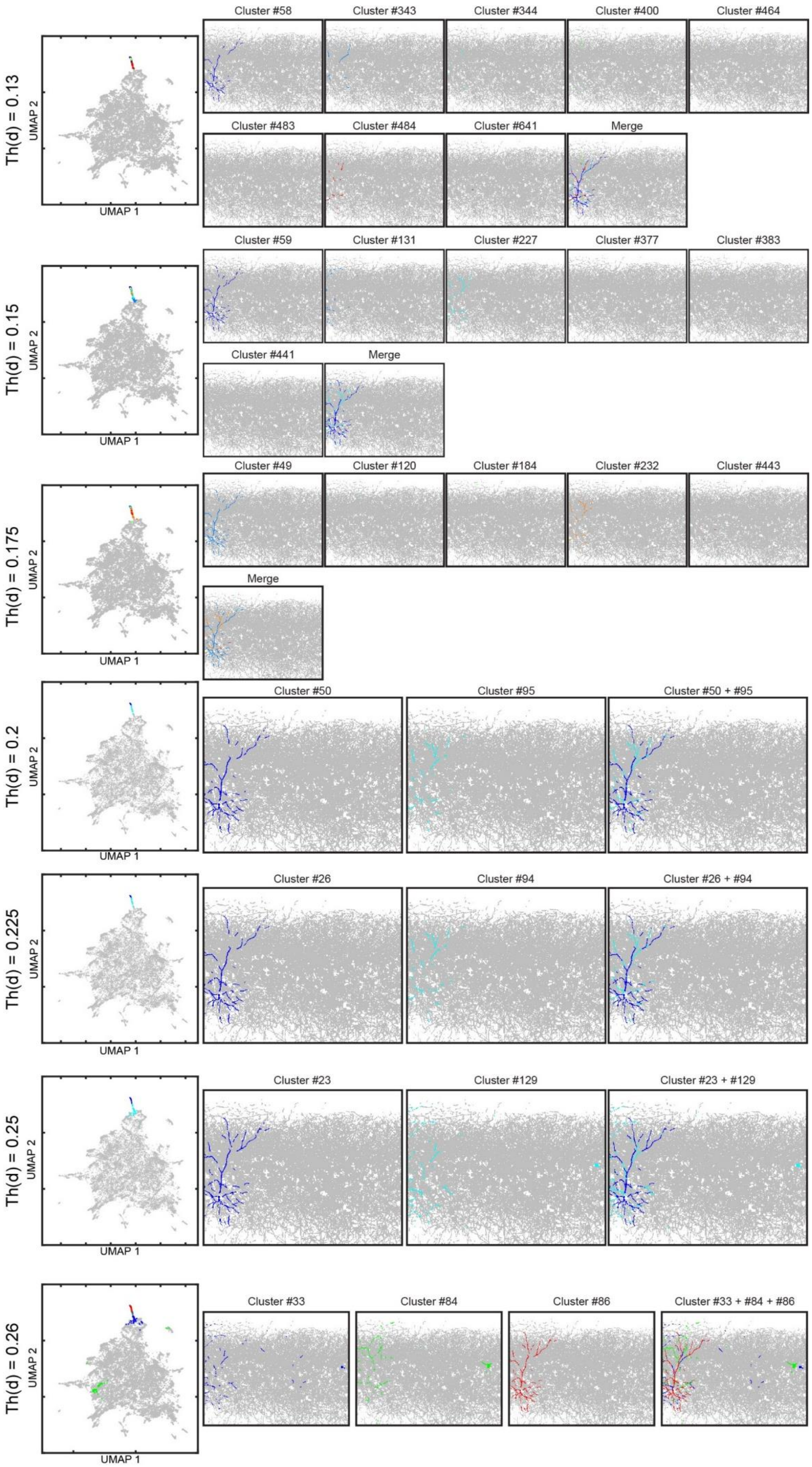

Th(d) = 0.13
UMAP 2
UMAP 1
Cluster #58
Cluster #343
Cluster #344
Cluster #400
Cluster #464
Cluster #483
Cluster #484
Cluster #641
Merge
Th(d) = 0.15
Cluster #59
Cluster #131
Cluster #227
Cluster #377
Cluster #383
Cluster #441
Merge
Th(d) = 0.175
Cluster #49
Cluster #120
Cluster #184
Cluster #232
Cluster #443
Merge
Th(d) = 0.2
Cluster #50
Cluster #95
Cluster #50 + #95
Th(d) = 0.225
Cluster #26
Cluster #94
Cluster #26 + #94
Th(d) = 0.25
Cluster #23
Cluster #129
Cluster #23 + #129
Th(d) = 0.26
Cluster #33
Cluster #84
Cluster #86
Cluster #33 + #84 + #86

Supplementary Fig. 9 | Investigation into a neuron represented by multiple clusters.

If *Th(d)* is too small, one neuron can split into multiple clusters. By using the UMAP plot we were able to occasionally identify neurons that were spread across multiple clusters. At our optimum threshold of 0.2 we detected that this example neuron contained fragments in clusters 50 and 95 (left panel). Therefore, we plotted all fragments belonging to each cluster separately (middle panels) and combined (right most panel). As we increase the *Th(d)* there are more fragments included within each cluster including false positives that clearly don't belong to the neuron. While as the *Th(d)* decreases there are more clusters associated with the fragments of the neuron. Note that UMAP distance does not necessarily reflect distance in the Euclidean space.

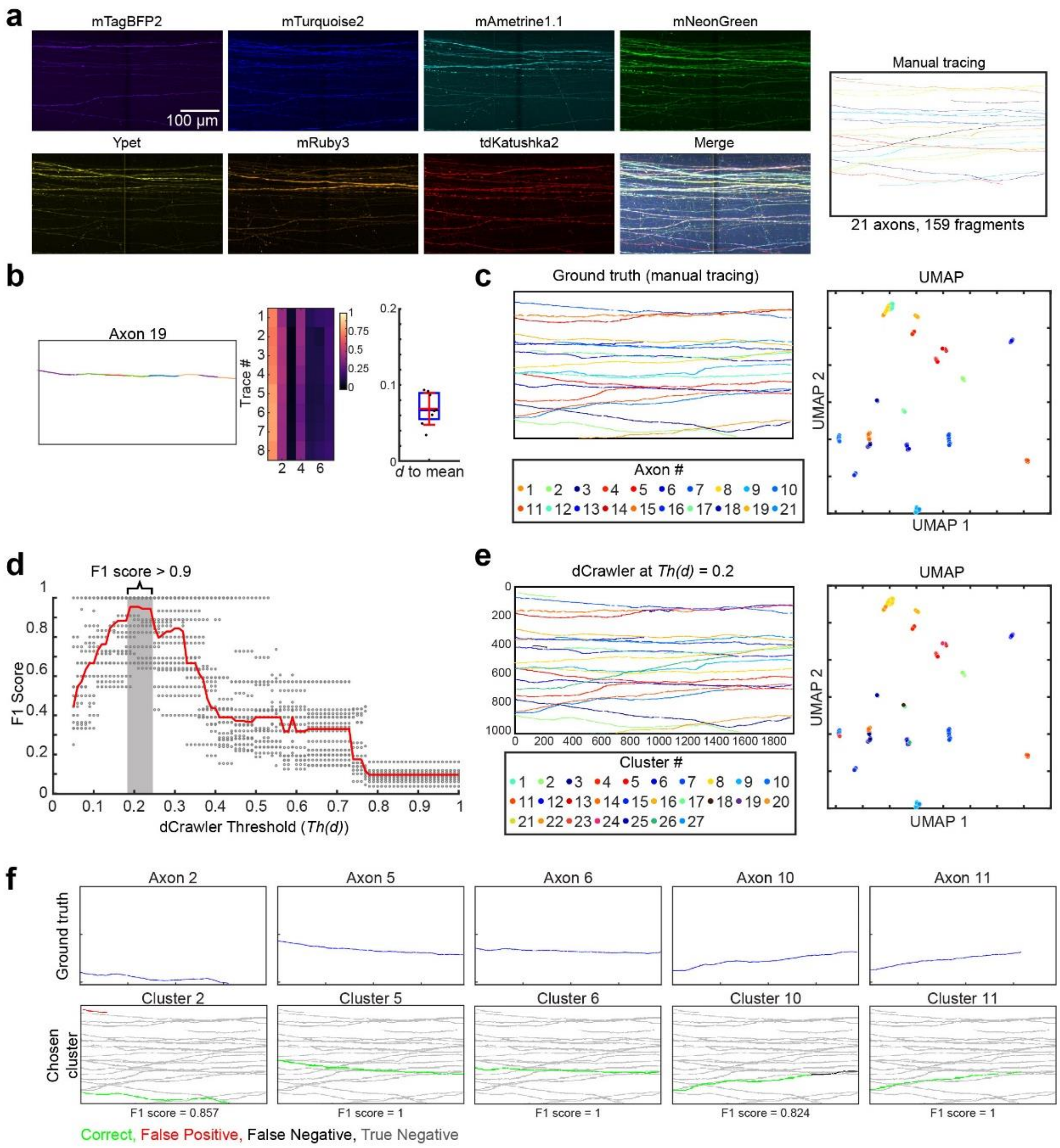

Supplementary Fig. 10 | Optimization for axonal reconstruction.

a, A 1 x 2 tiled image (A z-stacked image of 491.16 x 259.09 x 179.2 μm^3) of mitral and tufted cell axons were imaged at the lateral olfactory tract (left) and then manually traced using Neurolucida 360 (right). Mitral and tufted cell axons were labelled with 7-colour Tetbow. Mitral/tufted cell-specific Pcdh21-Cre mice and FLEX-tTA AAV were used to label mitral/tufted cells with AAV-TRE-XFP. The mouse was 7-week-old female. Image taken with a 20x objective lens.

b, A representative axon containing 8 fragments (left), their vector normalised colour vectors (middle), and the distance to the mean colour vector of the axon (right). See Supplementary Data 3 for all the results.

c, Ground truth of all the axons (manually traced). Left panel shows the location of fragments, while the right panel shows the location of these fragments in UMAP-reduced colour space.

d, The optimum threshold was calculated by running the dCrawler at a *d* ranging from 0.05 to 1. An F1 score was calculated for each axon at each *d* (gray dots), with the median F1 score for each *d* also displayed (red line). The optimum *d* was calculated to be 0.2.

e, The traces after clustering at the the optimum *Th(d)*, shown in physical space (a z-stacked image, left), and in UMAP-reduced colour space (right).

f, Representative neurons (blue lines, top row) paired to their best cluster (bottom row). Traces in both the neuron and the cluster are considered correct (green), those only in the cluster are a false positive (red), those in the neuron only as a false negative (black), and those in neither the neuron or cluster as a true negative (grey). See Supplementary Data 4 for all the results.

Source data are provided as a Source Data file.

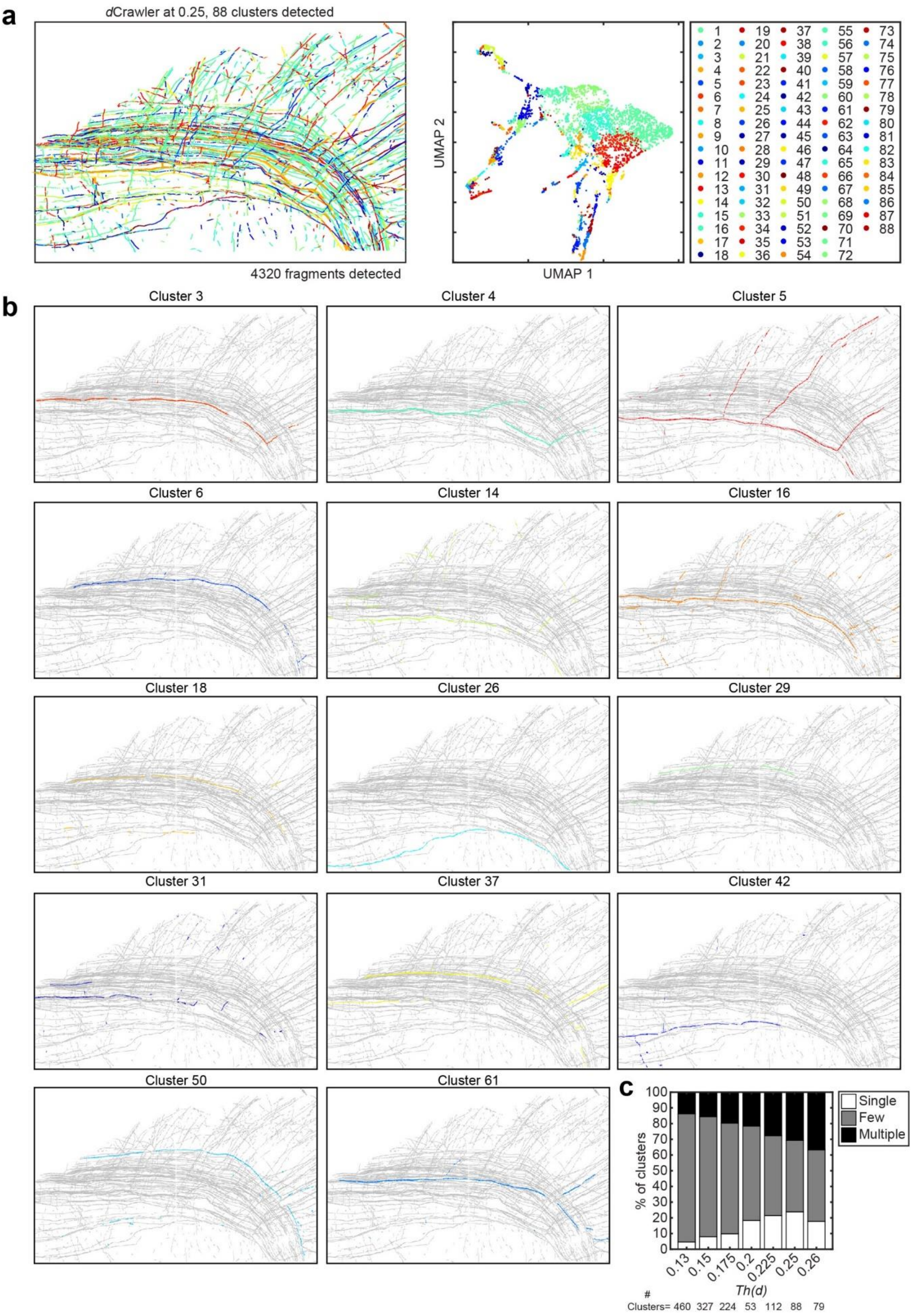

Supplementary Fig. 11 | Evaluation of mitral/tufted cell axons identified with QDyeFinder

a, All fragments detected automatically by Neurolucida360 in physical (left) and UMAP-reduced (right) space. Fragments are colour coded according to their cluster obtained when *Th(d)* = 0.25.

b, 14 clusters identified that seems to cover axons for a single neuron at *Th(d)* = 0.25.

c, Classification of dCrawler clusters at a range of *Th(d)* for mitral/tufted cell axon data. Clusters were grouped by whether a cluster contained a single neuron, multiple neurons, or few fragments. The best results were with *Th(d)* = 0.25.

Source data are provided as a Source Data file.

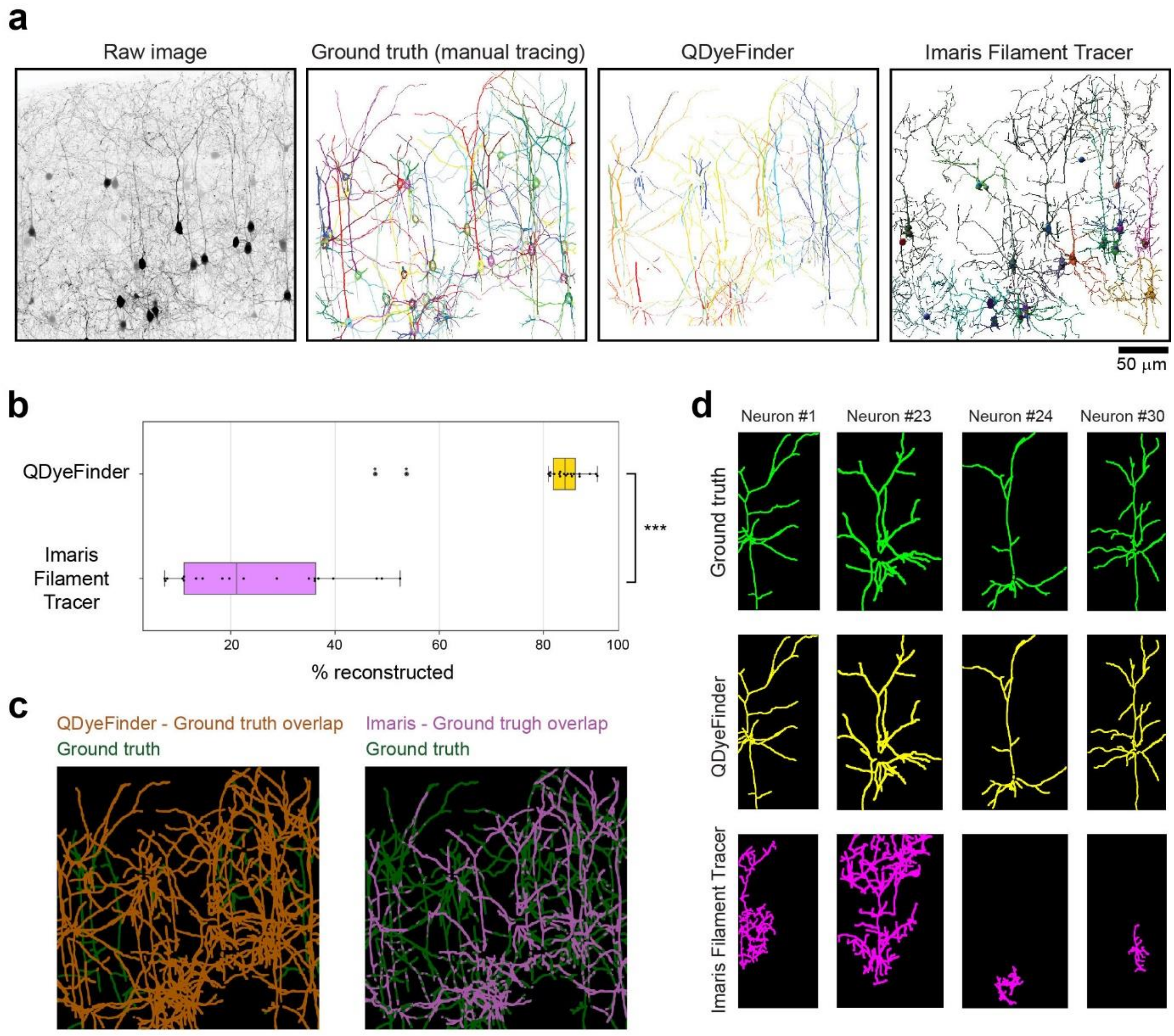

Supplementary Fig. 12 | Comparison with existing auto-tracing software.

a, Automated neuron reconstruction with QDyeFinder and Imaris 10 Filament tracer. The ground truth was prepared by manual tracing with Neurolucida 360 as described in Fig. 5.

b, Quantitative comparison between QDyeFinder and Imaris 10 Filament tracer. Percent reconstructed indicates the area of intersection out of the area of the ground truth. Box plots indicate median ± IQR (20 neurons). As we could only reconstruct 20 neurons in Imaris, we compared the 20 neurons. $p^{***} < 0.001$ (Mann-Whitney U-test)

c, Successfully reconstructed neurites (indicated in **b**) are shown.

d, Some examples of single neuron reconstructions.

Reconstruction data in swc format are available at Zenodo (https://zenodo.org/records/11482026). Source data are provided as a Source Data file.

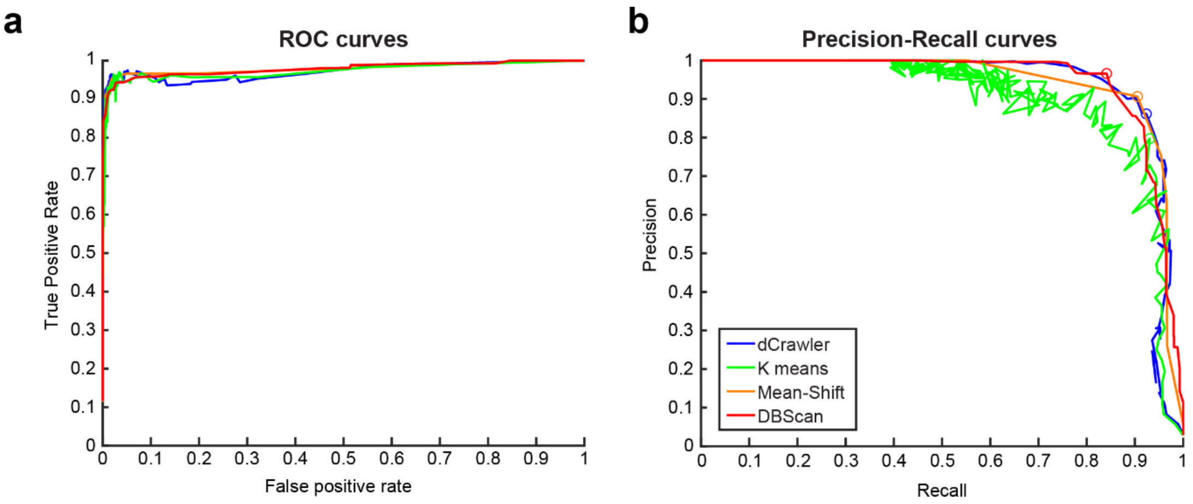

c

Clustering algorithm	Optimum F1 Mean	Optimum F1 Median	Optimum setting value Mean	Optimum setting value Median	Area Under the Curve (AUC) ROC	Area Under the Curve (AUC) PR
dCrawler	0.882	0.971	d = 0.170	d = 0.200	0.974	0.942
Mean Shift clustering	0.876	0.982	b = 0.050	b = 0.060	0.980	0.949
k-means clustering	0.862	0.941	k = 46	k = 42	0.972	0.890
DBSCAN	0.880	0.941	ε = 0.900	ε = 0.900	0.979	0.946

Supplementary Fig. 13 | Comparison of clustering algorithms in QDyeFinder.

a, ROC (Receiver Operating Characteristic) curves showing that the performance of the algorithms is broadly similar.

b, PR (Precision Recall) curves provide better insight to the algorithms performance as there is a class imbalance (many more true negatives that true positives). In this dataset k-means (green line) performs the worst while the remaining three are broadly similar with the selection of appropriate parameters being the most important step.

c, Summary of the optimum parameters and performance under the best conditions.

Source data are provided as a Source Data file.

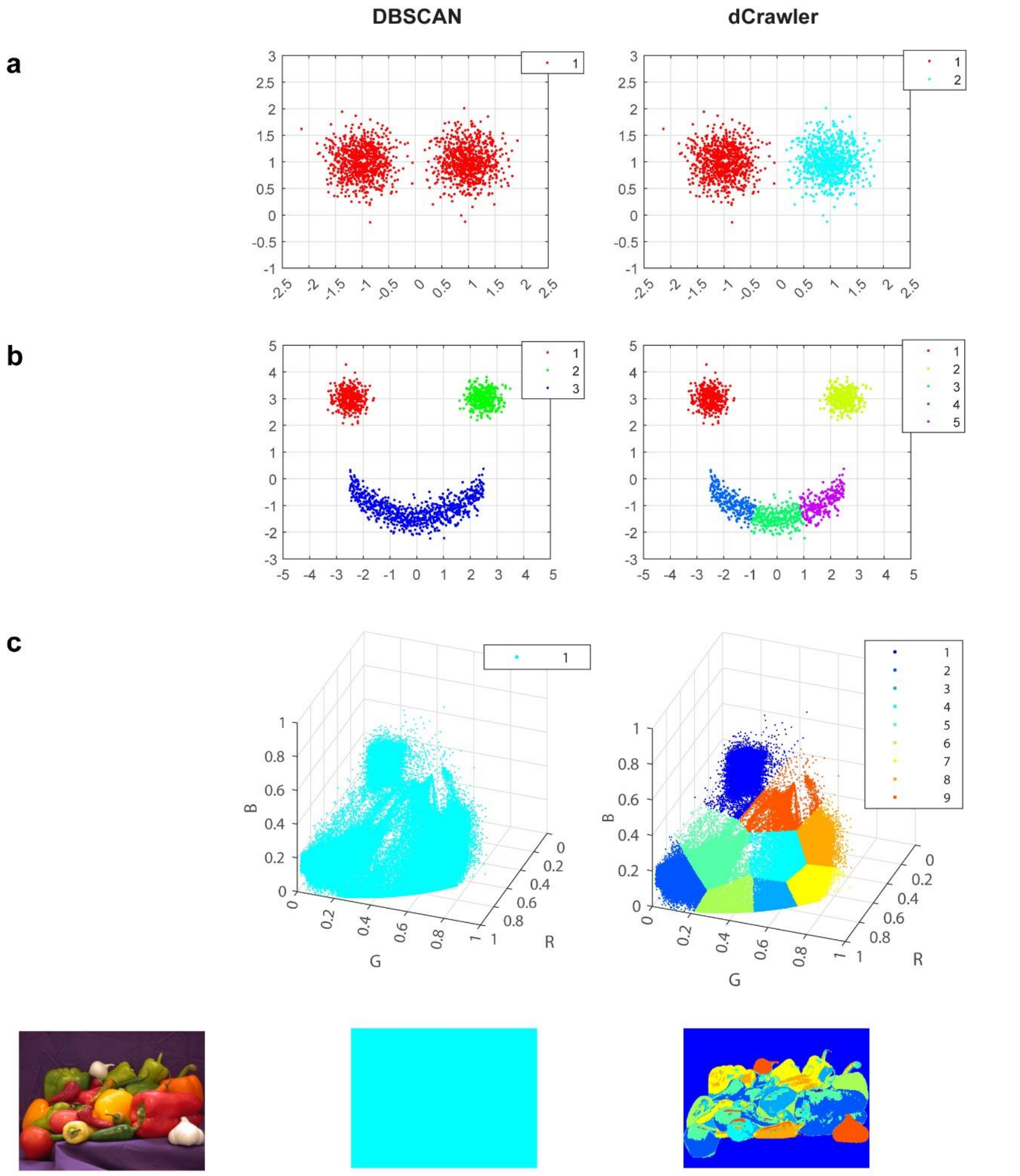

Supplementary Fig. 14 | Comparison between DBSCAN and dCrawler.

Both DBSCAN and dCrawler are optimised for different situations.

a, A situation where dCrawler outperforms DBSCAN. When two clusters are close or adjacent, DBSCAN will incorporate them into a single cluster by its nature (left panel). DBSCAN only works well when the boundaries between clusters are clear. Since dCrawler's threshold is tied to the putative centroid, the two clusters remain separate.

b, A situation where DBSCAN outperforms dCrawler. In this case, the bottom cluster (the mouth) isn’t uniform in its distribution but is successfully grouped into 1 cluster by DBSCAN. dCrawler identifies 3 separate clusters instead of 1 in reality.

c, Real-world example of clustering by colours using the MATLAB example image peppers.png (https://jp.mathworks.com/help/images/reduce-the-number-of-colors-in-an-image.html, used with permission from The MathWorks, Inc. © 1994-2021 The MathWorks, Inc.). DBSCAN is unable to identify individual colours as the points are spread in a spectral manner. While dCrawler is able to handle this scenario and identify 9 clusters/colours.

Tracing strategy	**Manual tracing based on physical continuity**	**Auto-tracing based on physical continuity**	**Multicolour (Brainbow/Tetbow)**
Examples of software	Aivia,<br>Amira,<br>HortaCloud,<br>NeuTube,<br>Neurolucida,<br>Simple Neurite Tracer,<br>Vaa3D	Arivis Vision4D,<br>Imaris Filament Tracer,<br>Neurolucida 360,<br>Vaa3D	QDyeFinder,<br>Sümbül et al
Prior parameter optimization	Not required<br>(but requires training of human skills)	Required<br>(parameter optimization or training dataset for deep learning)	Required<br>(clustering parameters are needed, e.g., Th(d))
Speed	Slow	High	High
Accuracy for long-neurites	High<br>(gradually decrease)	Low<br>(gradually decrease)	High<br>(as long as the colour hue is consistent)
Accuracy for densely labelled circuits	High<br>(as long as neurites are visually discernible)	Low	High<br>(but limited to ~100 neurons when 7 XFPs are used)
Tracing broken neurites / across images	No	No	Yes

Supplementary Table 1 | Comparison of different tracing software for neurite reconstruction based on light microscope images.

Supplementary Note 1 | Conversion of Imaris files to SWC format

In this study, we mainly used Neurolucida 360 to automatically detect neurite fragments. However, Imaris Filament Tracer is also commonly used for neurite detection. Data format of Imaris files (.hoc) are different from .swc format commonly used for tracing software. The conversion is done with the SWC exporter plugin (https://github.com/Elsword016/Swc-plugins-for-Imaris-10.git) written for Imaris 10 and Python3.7.

Installation:

Place the python scripts in the folder with the ImarisLib library folders (default is C:/ProgramFiles/Bitplane/Imaris10/XT/python3/) and then run it from the Imaris window and follow the prompts on the GUI.

Procedure:

1 . Open the image volume with filaments.

2. Select the filaments you want to export.

3. Go to Image Processing > SWC Exporter

4. Select the save location using the dialog. The plugin will automatically save each of the filaments as separate swc files as well as one combined swc.

Supplementary Note 2 | dCrawler description

Step 1: Crawl through clusters

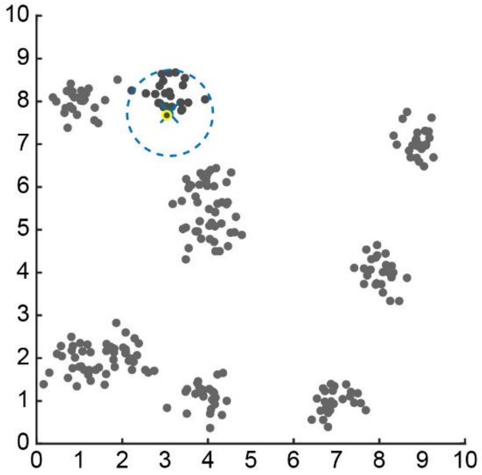

Begining at your first point, establish cluster 1 as only this single point (blue), and use its position as the centroid for the cluster (blue cross).
Then find the closet point (yellow), and determine, if it is within the threshold (dashed circle)

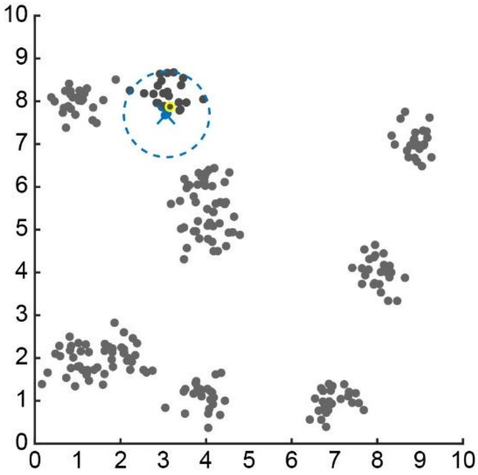

If the next point is within the threshold, then we include it into our cluster. And update the centroid position. (NB the centroid calcuation can also use a weighted mean if required).

We then search to see if the next point is within the threshold distance to the the cluster

We keep searching through until there are no more new points within the threshold distance to the centroid

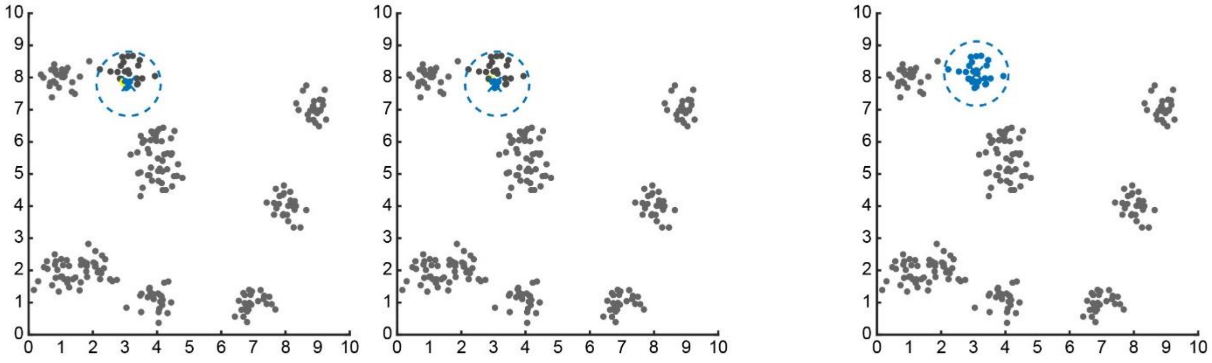

However, sometimes it is possible for points to leave the cluster if the distance from the updated centroid position is larger than the threshold (green)

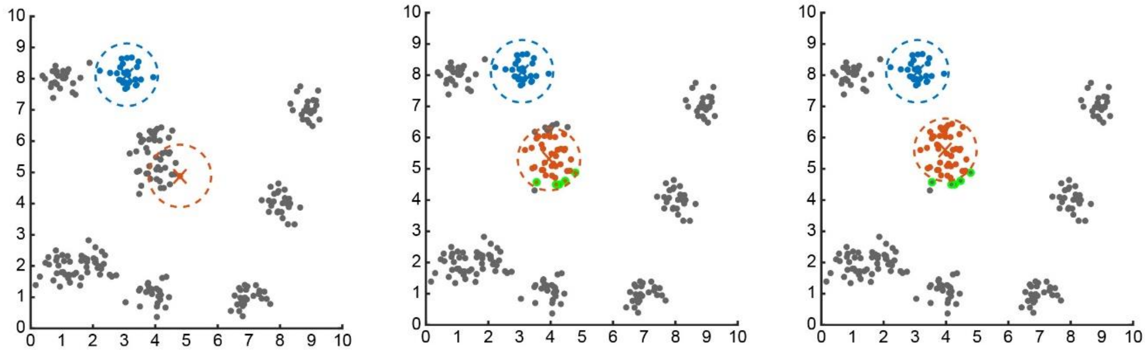

Additionally, for the first sweep, if closest point is within the threshold but already belongs to a different cluster they are ignored (black outline)

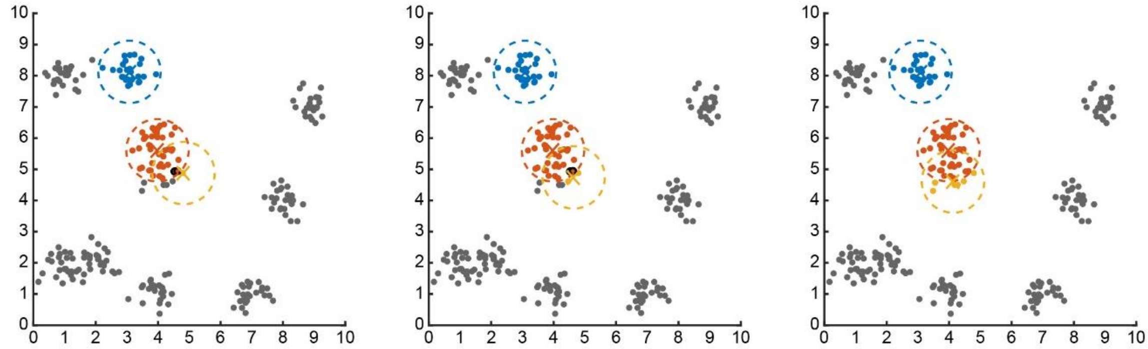

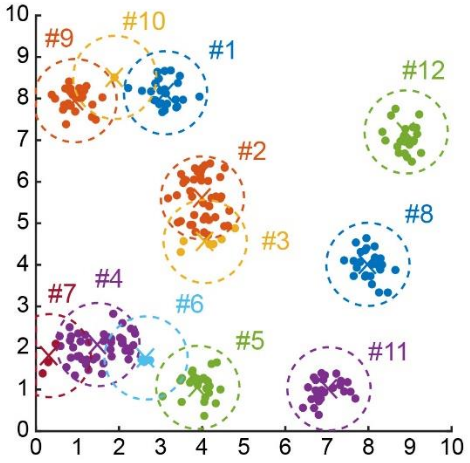

This generates our first pass of the crawler. However, there is a primacy bias which we will need to correct for. (That is the clusters that are generated first will include points that are closer a later generated cluster)

Flowchart for the Crawler

For points i=1 to n

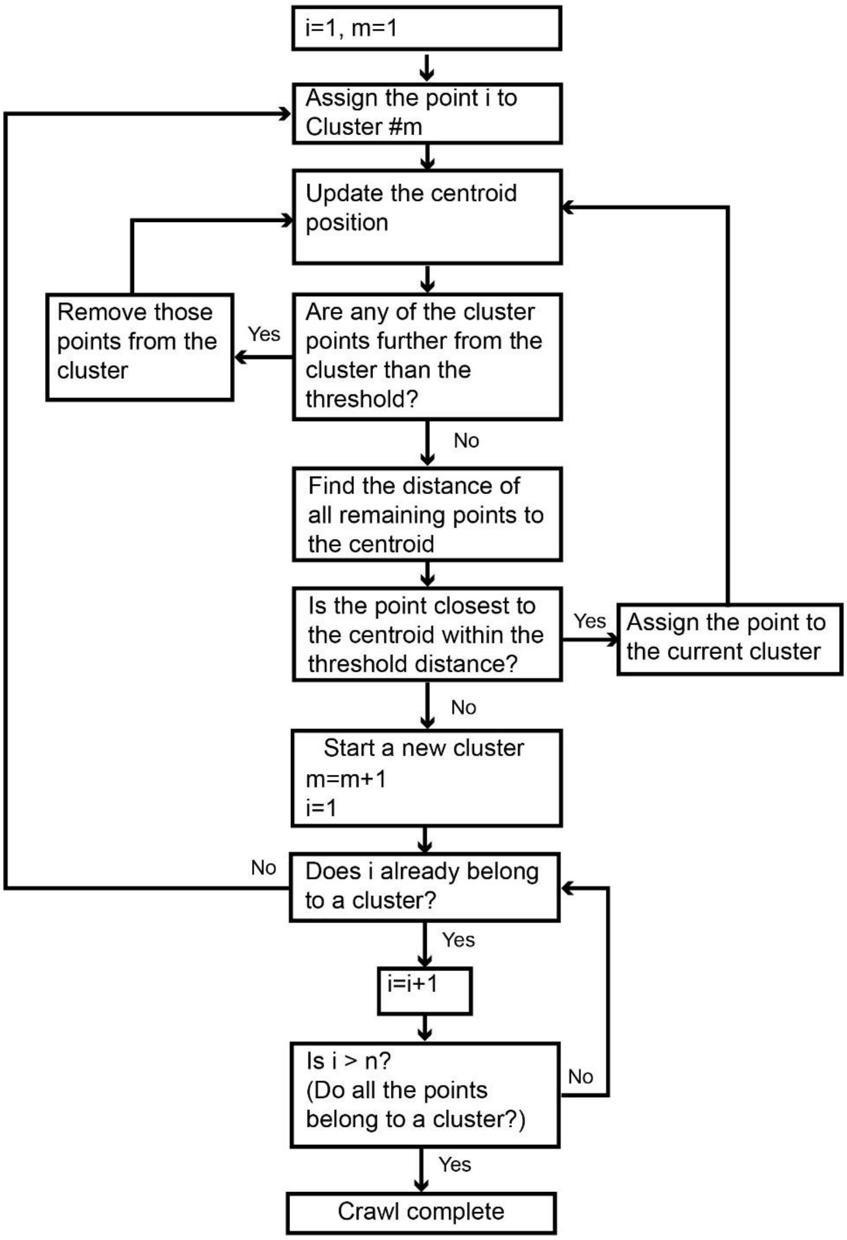

Step 2: Adjust Clusters

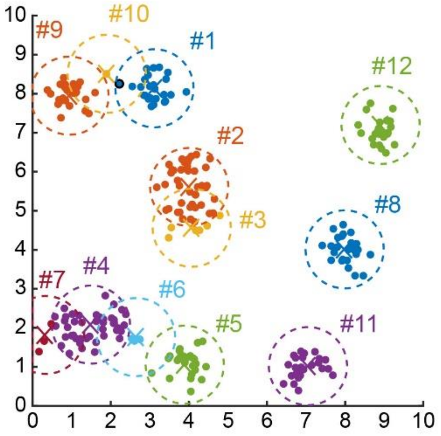

So now we go through each point and assign each point to the cluster with the closest centroid (e.g. black outline).Then we update the centroid positions after each adjustment.
NB For a fast implementation of the program. Instead of restarting the loop after each adjustment. I keep checking the remaining points. The adjustment is only considered complete when the final loop has no adjustments.

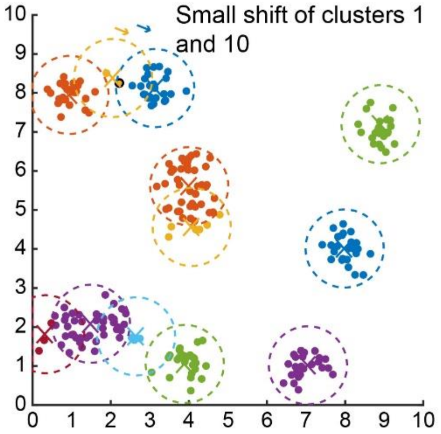

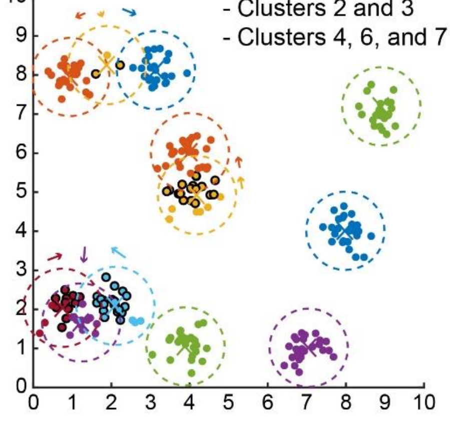

Flowchart for Adjusting Clusters

For points i=1 to n

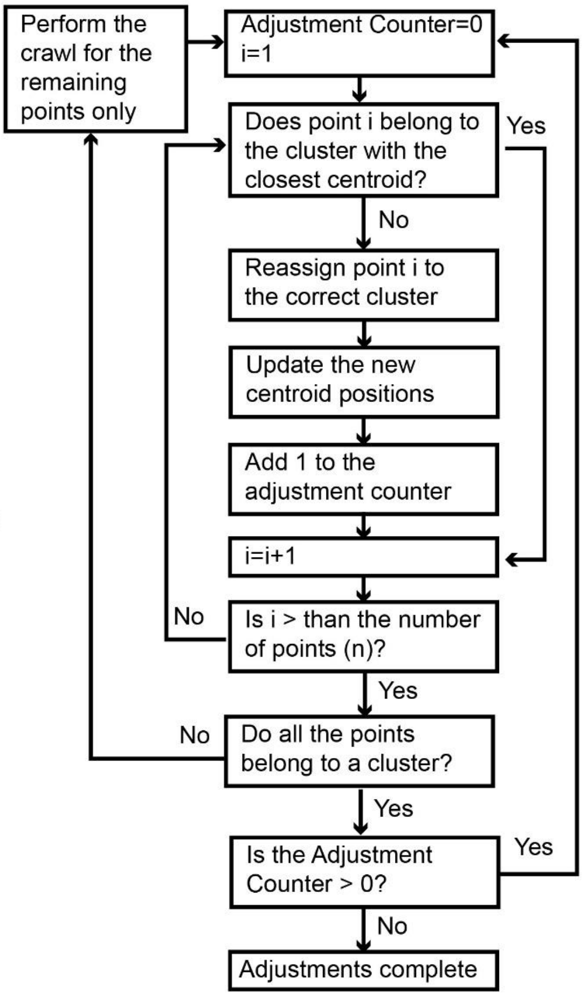

Step 3: Merge clusters

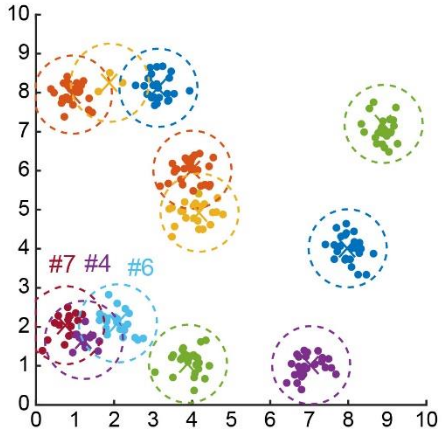

After the adjustment it looks like Clusters #2 and #3 are resolved. However clusters #4, #6, and #7 (esp #4 and #7) look like they should actually be the same cluster.

So now to evaluate the closeness of clusters we measure the distance between the centroids to evaluate this. Then we redo the crawler and make adjusstments if necessary.

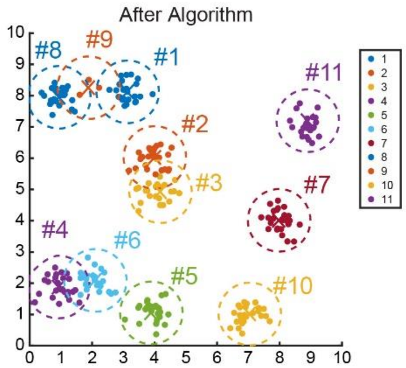

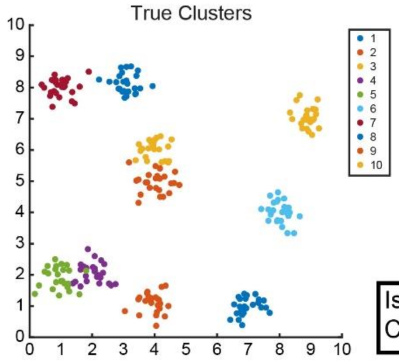

Flowchart for Merging Clusters

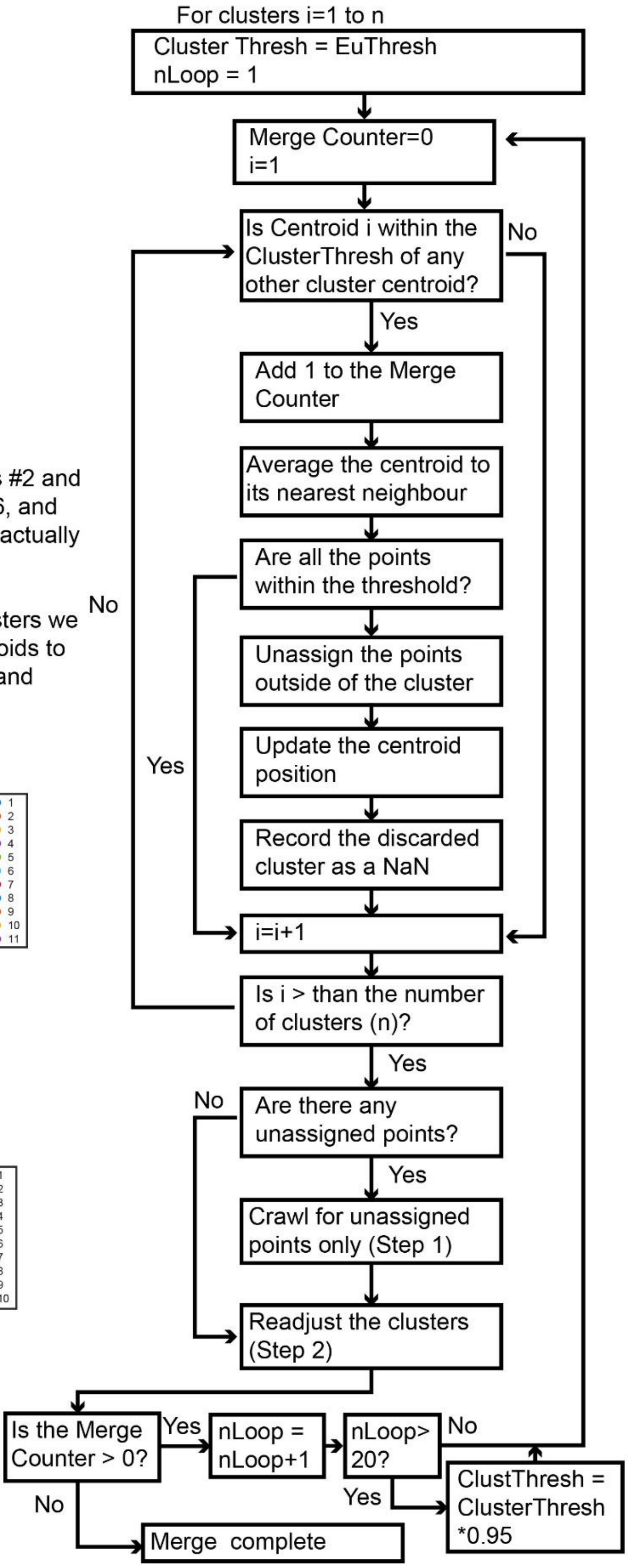

Supplementary Data 1 | Evaluation of colour consistency in dendrites
Relevant fragments (left), the vector normalised colour vector of each trace (middle), and the Euclidean distance to the centroid colour vectr for the neuron (right). On the middle, *x* axis indicate channels and *y* axis shows the traces. On the right, red bars indicates mean ± SD and blue box plots indicate median ± IQR of *d* to the centroid for all fragments. See also Fig. 5b.

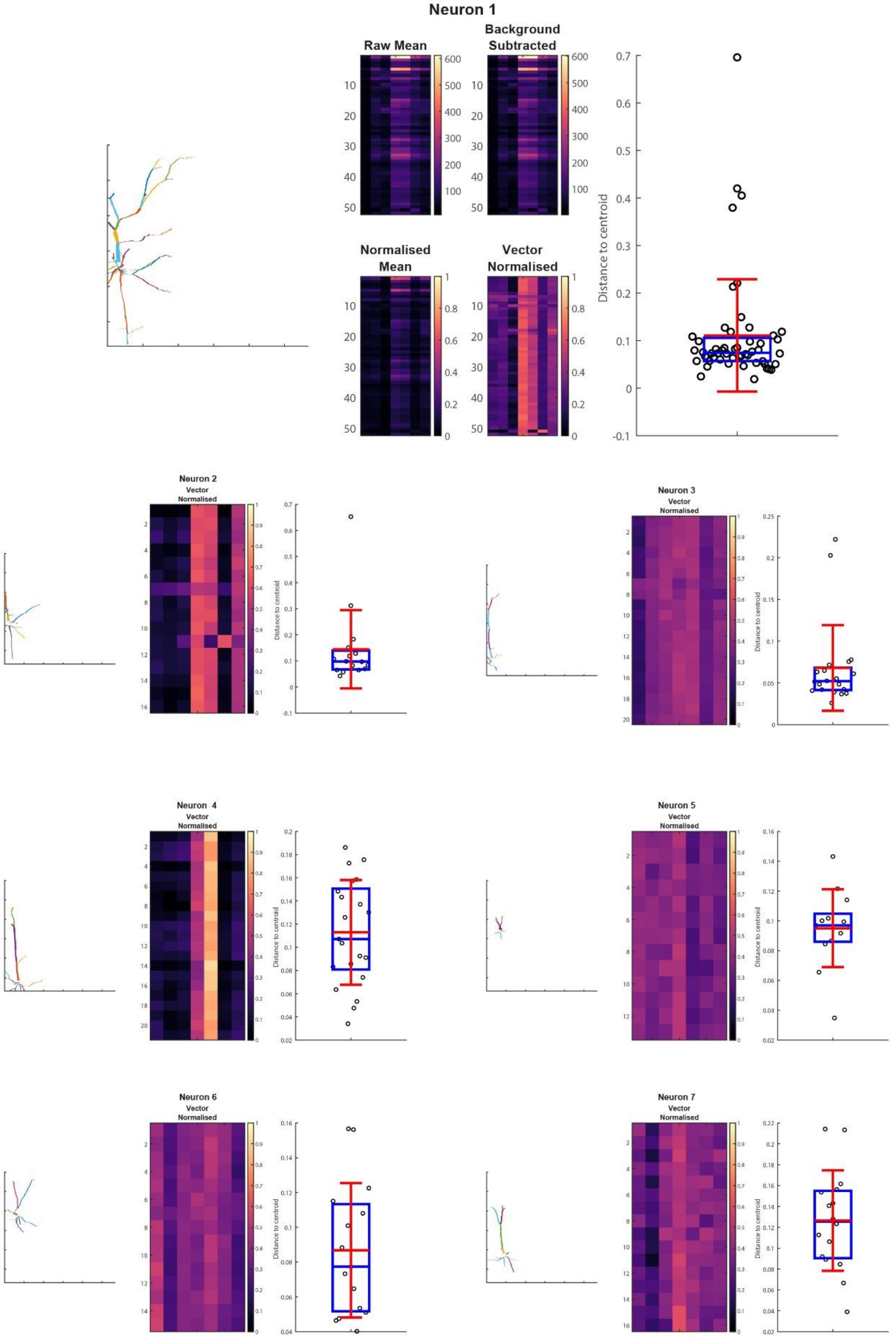

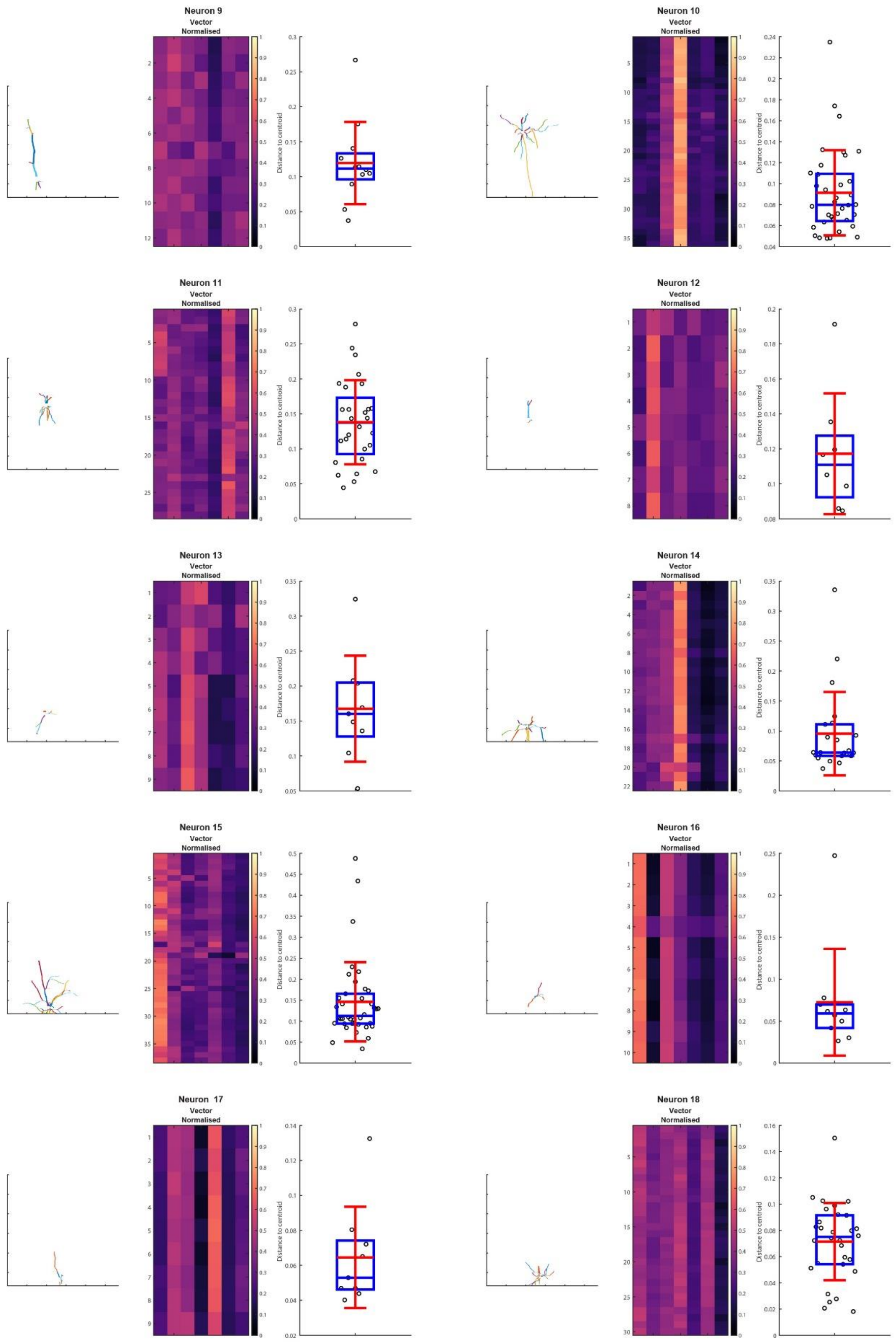
Neuron 9
Vector
Normalised
Distance to centroid
Neuron 10
Vector
Normalised
Distance to centroid
Neuron 11
Vector
Normalised
Distance to centroid
Neuron 12
Vector
Normalised
Distance to centroid
Neuron 13
Vector
Normalised
Distance to centroid
Neuron 14
Vector
Normalised
Distance to centroid
Neuron 15
Vector
Normalised
Distance to centroid
Neuron 16
Vector
Normalised
Distance to centroid
Neuron 17
Vector
Normalised
Distance to centroid
Neuron 18
Vector
Normalised
Distance to centroid

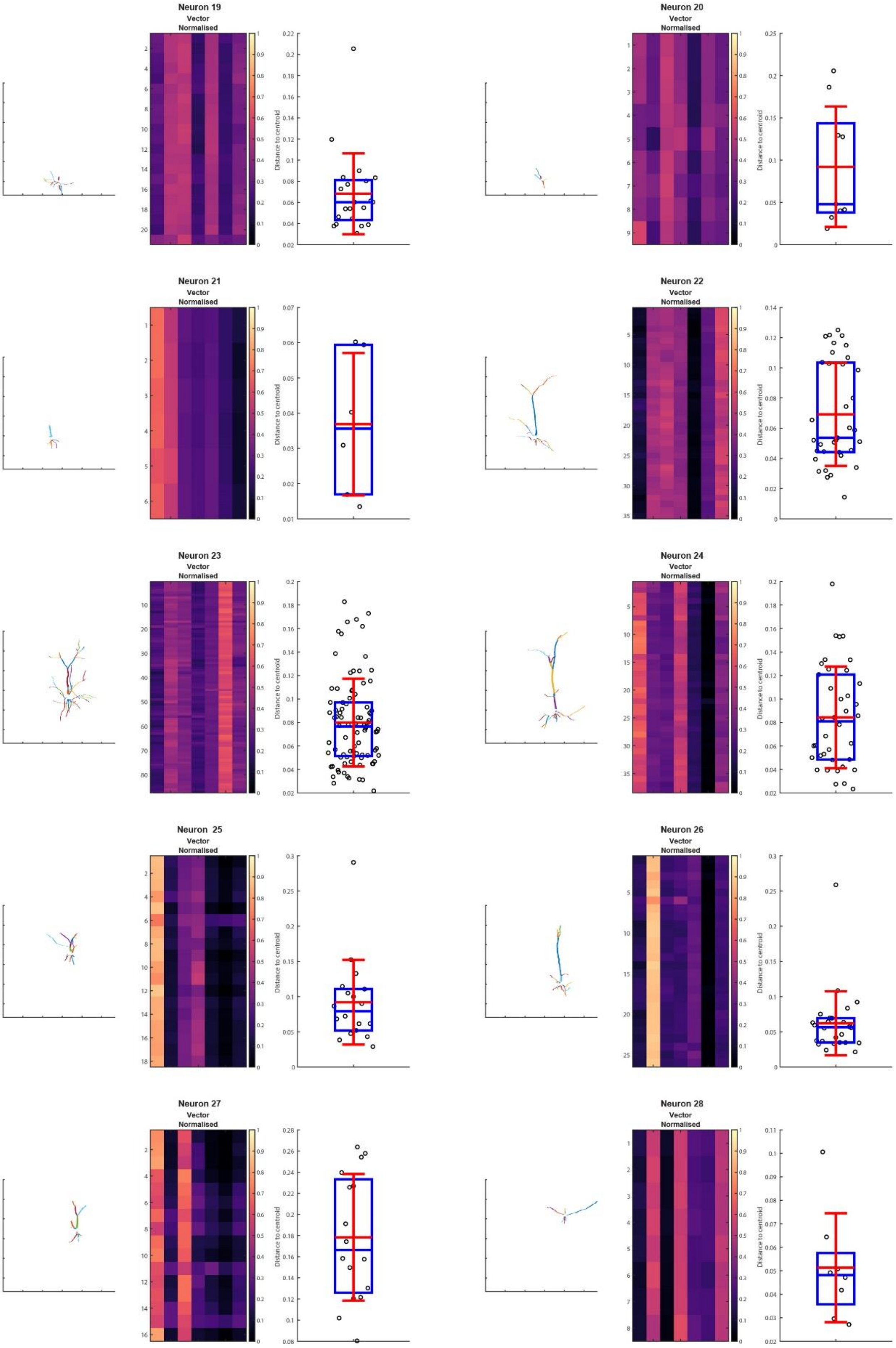
Neuron 19
Vector
Normalised
Distance to centroid
Neuron 20
Vector
Normalised
Distance to centroid
Neuron 21
Vector
Normalised
Distance to centroid
Neuron 22
Vector
Normalised
Distance to centroid
Neuron 23
Vector
Normalised
Distance to centroid
Neuron 24
Vector
Normalised
Distance to centroid
Neuron 25
Vector
Normalised
Distance to centroid
Neuron 26
Vector
Normalised
Distance to centroid
Neuron 27
Vector
Normalised
Distance to centroid
Neuron 28
Vector
Normalised
Distance to centroid

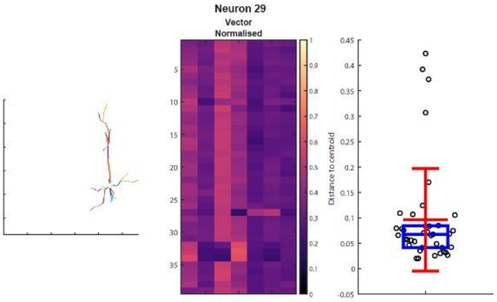
Neuron 29
Vector
Normalised
Distance to centroid

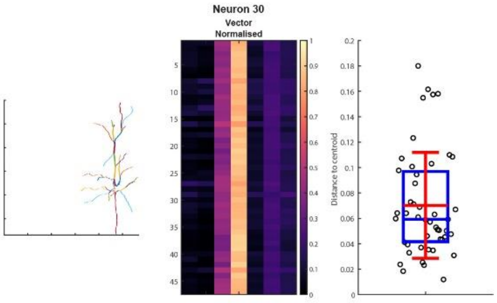
Neuron 30
Vector
Normalised
Distance to centroid

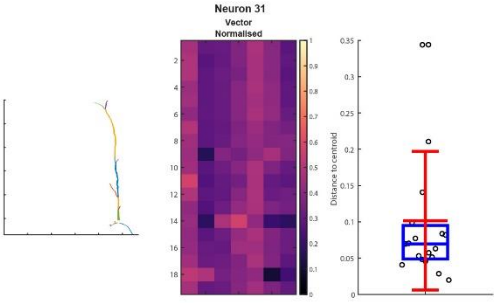
Neuron 31
Vector
Normalised
Distance to centroid

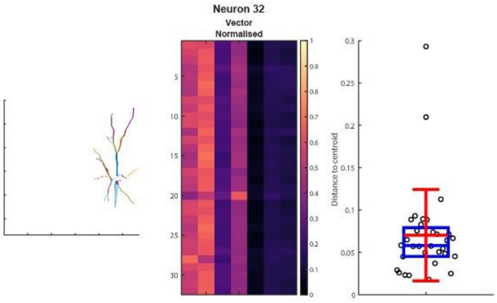
Neuron 32
Vector
Normalised
Distance to centroid

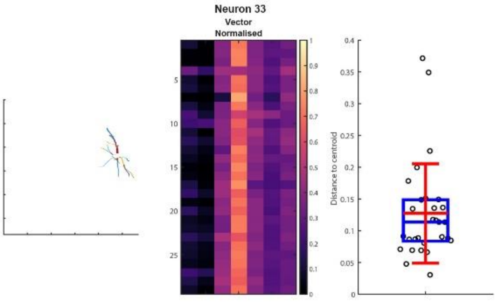
Neuron 33
Vector
Normalised
Distance to centroid

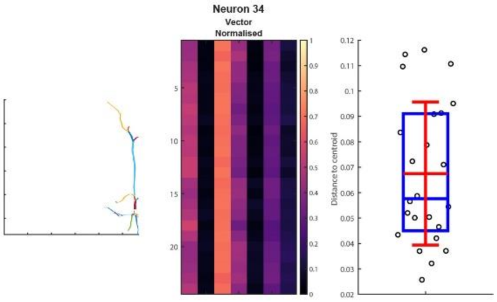
Neuron 34
Vector
Normalised
Distance to centroid

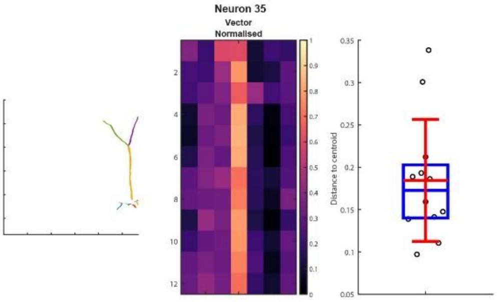
Neuron 35
Vector
Normalised
Distance to centroid

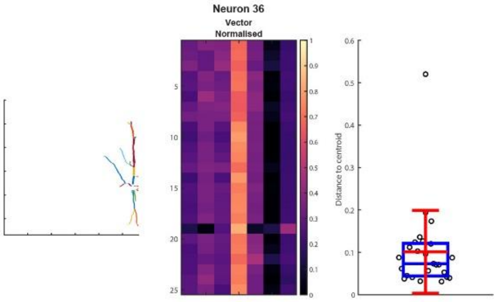
Neuron 36
Vector
Normalised
Distance to centroid

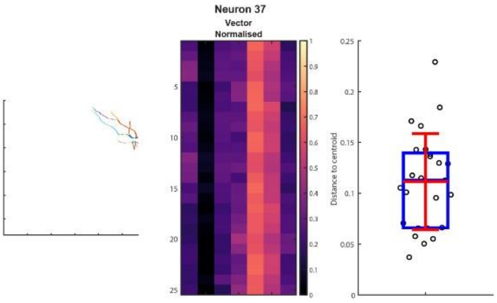
Neuron 37
Vector
Normalised
Distance to centroid

Supplementary Data 2 | Comparison of dCrawler clusters vs. ground truth for dendrites
X and y axes show pixel numbers of the images. See also Fig. 5f.

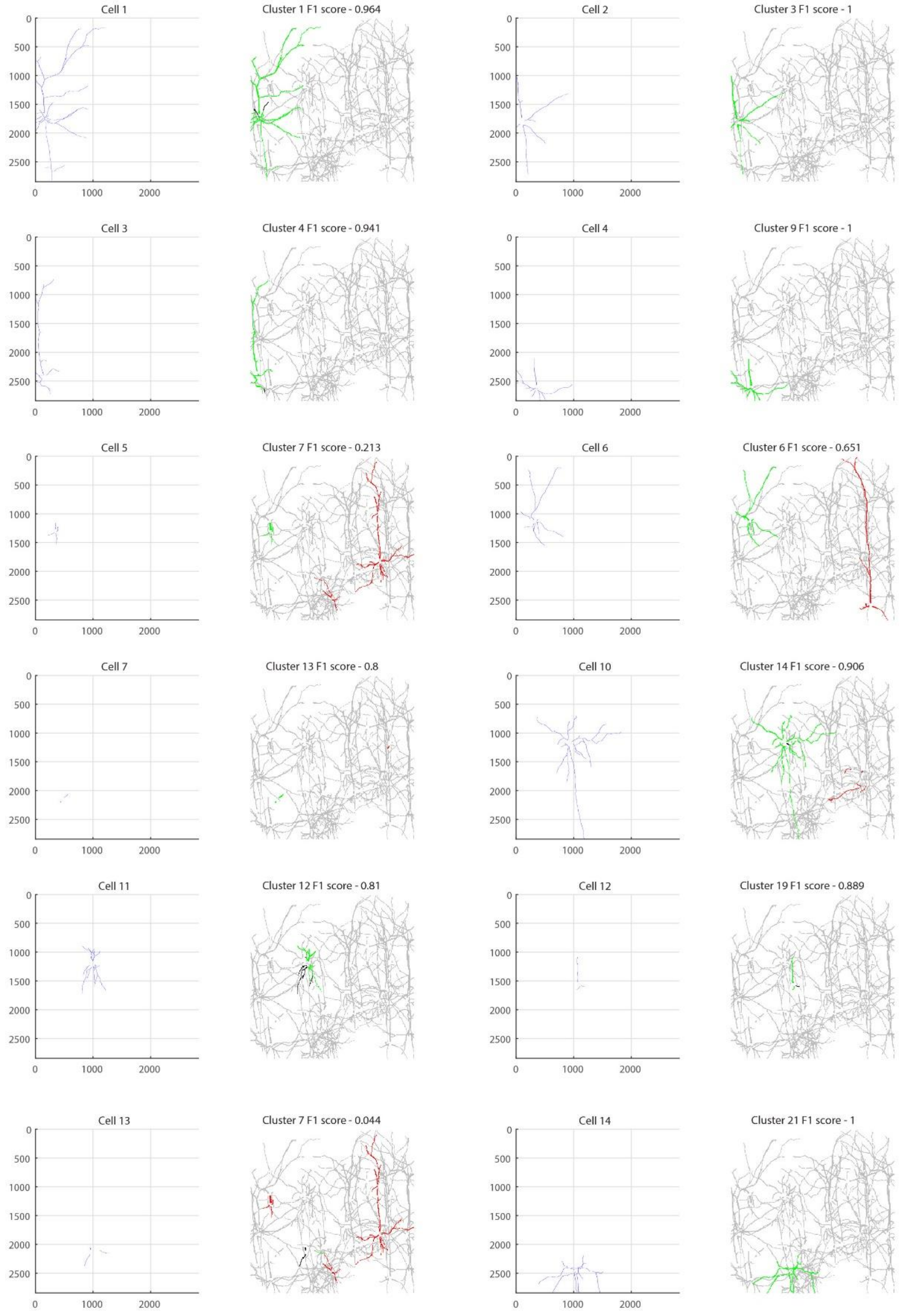

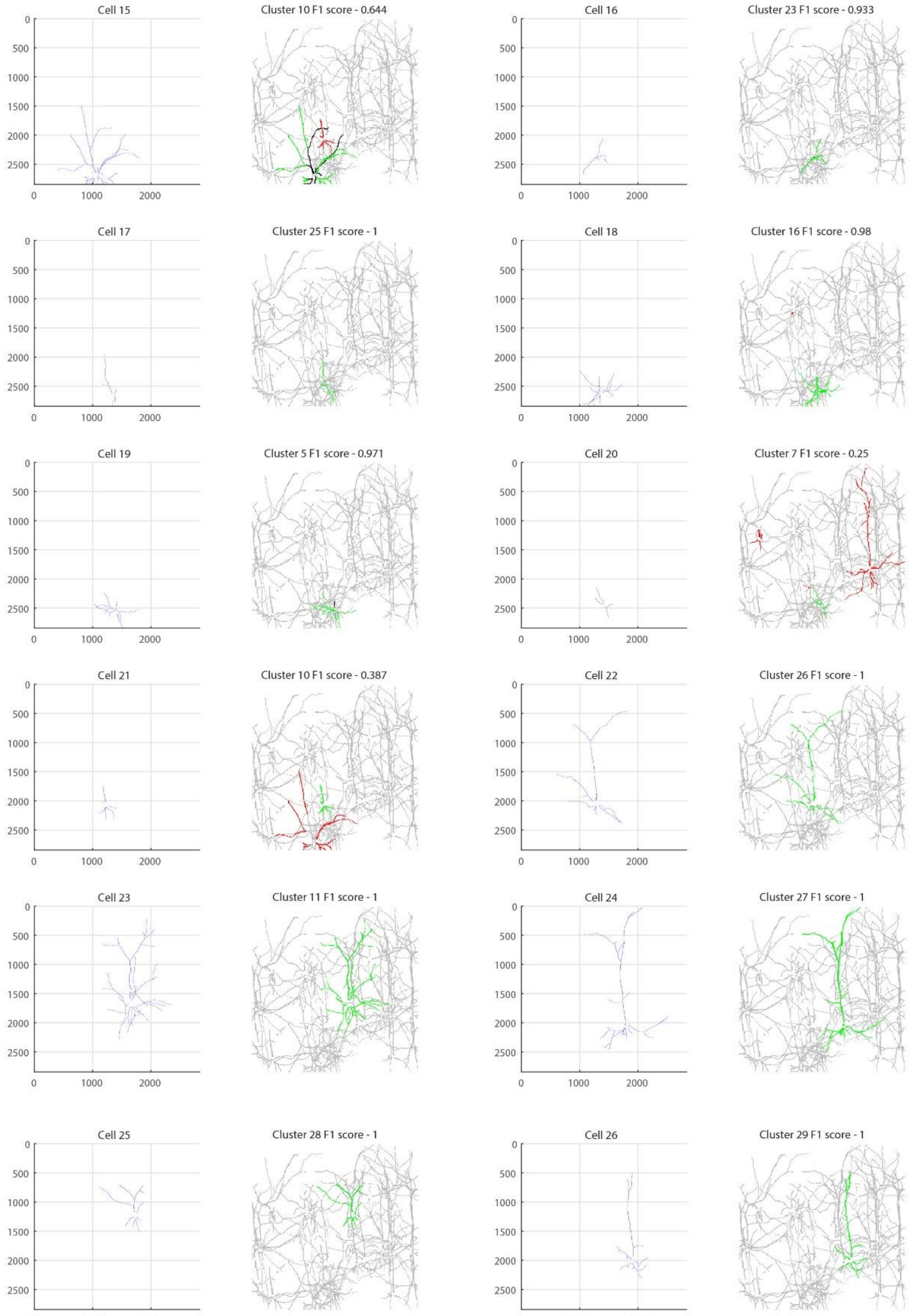
Cell 15
Cluster 10 F1 score - 0.644
Cell 16
Cluster 23 F1 score - 0.933
Cell 17
Cluster 25 F1 score - 1
Cell 18
Cluster 16 F1 score - 0.98
Cell 19
Cluster 5 F1 score - 0.971
Cell 20
Cluster 7 F1 score - 0.25
Cell 21
Cluster 10 F1 score - 0.387
Cell 22
Cluster 26 F1 score - 1
Cell 23
Cluster 11 F1 score - 1
Cell 24
Cluster 27 F1 score - 1
Cell 25
Cluster 28 F1 score - 1
Cell 26
Cluster 29 F1 score - 1

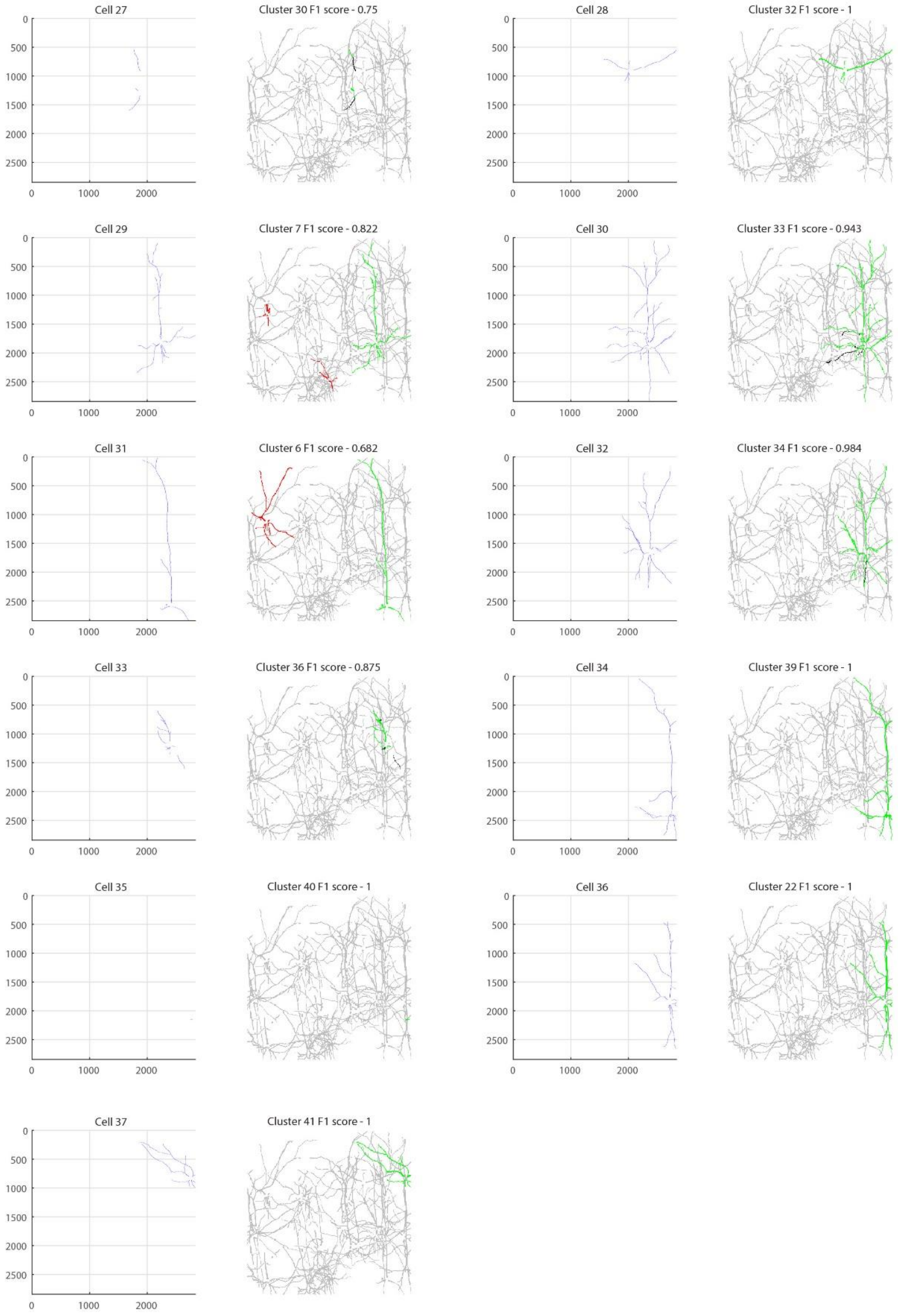

Cell 27
Cluster 30 F1 score - 0.75
Cell 28
Cluster 32 F1 score - 1
Cell 29
Cluster 7 F1 score - 0.822
Cell 30
Cluster 33 F1 score - 0.943
Cell 31
Cluster 6 F1 score - 0.682
Cell 32
Cluster 34 F1 score - 0.984
Cell 33
Cluster 36 F1 score - 0.875
Cell 34
Cluster 39 F1 score - 1
Cell 35
Cluster 40 F1 score - 1
Cell 36
Cluster 22 F1 score - 1
Cell 37
Cluster 41 F1 score - 1
0
500
1000
1500
2000
2500
0
1000
2000

Supplementary Data 3 | Evaluation of colour consistency in axons

See legends to Supplemnetary Data 1 for axes and labels. See also Supplementary Fig. 10b.

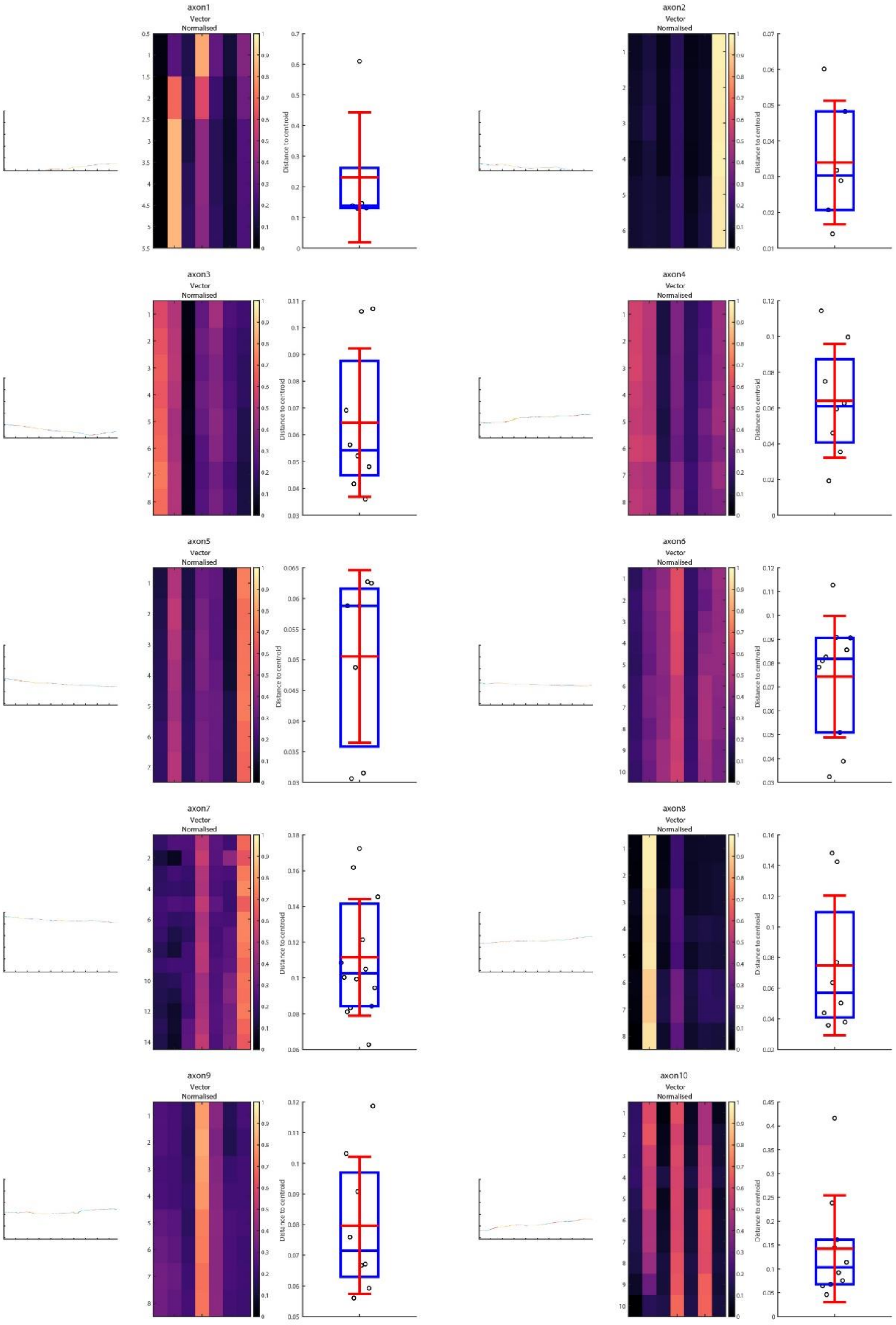

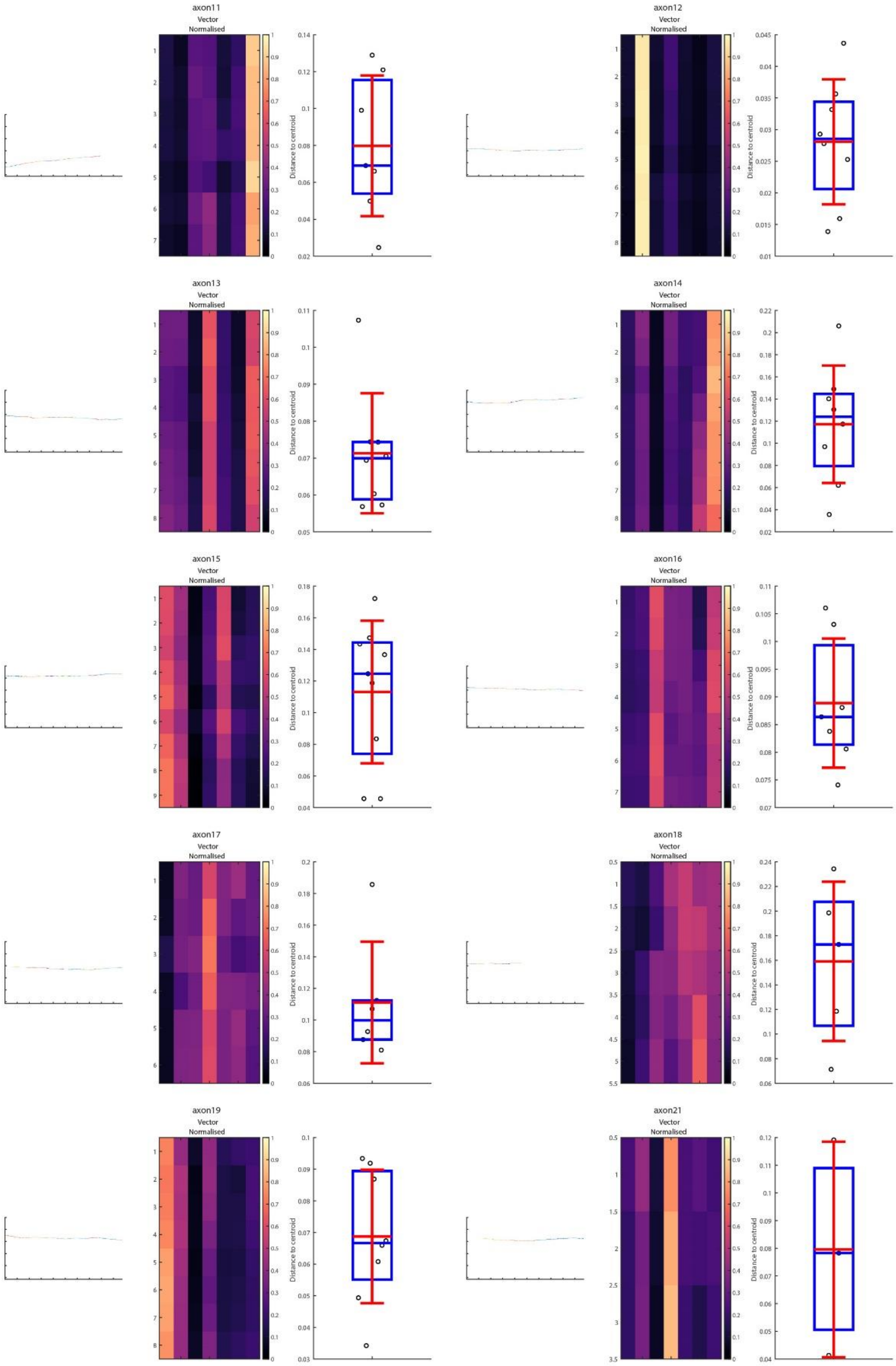
axon11
Vector
Normalised
Distance to centroid
axon12
Vector
Normalised
Distance to centroid
axon13
Vector
Normalised
Distance to centroid
axon14
Vector
Normalised
Distance to centroid
axon15
Vector
Normalised
Distance to centroid
axon16
Vector
Normalised
Distance to centroid
axon17
Vector
Normalised
Distance to centroid
axon18
Vector
Normalised
Distance to centroid
axon19
Vector
Normalised
Distance to centroid
axon21
Vector
Normalised
Distance to centroid

Supplementary Data 4 | Comparison of dCrawler clusters vs. ground truth for axons X and y axes show pixel numbers of the images. See also Supplementary Fig. 10f.

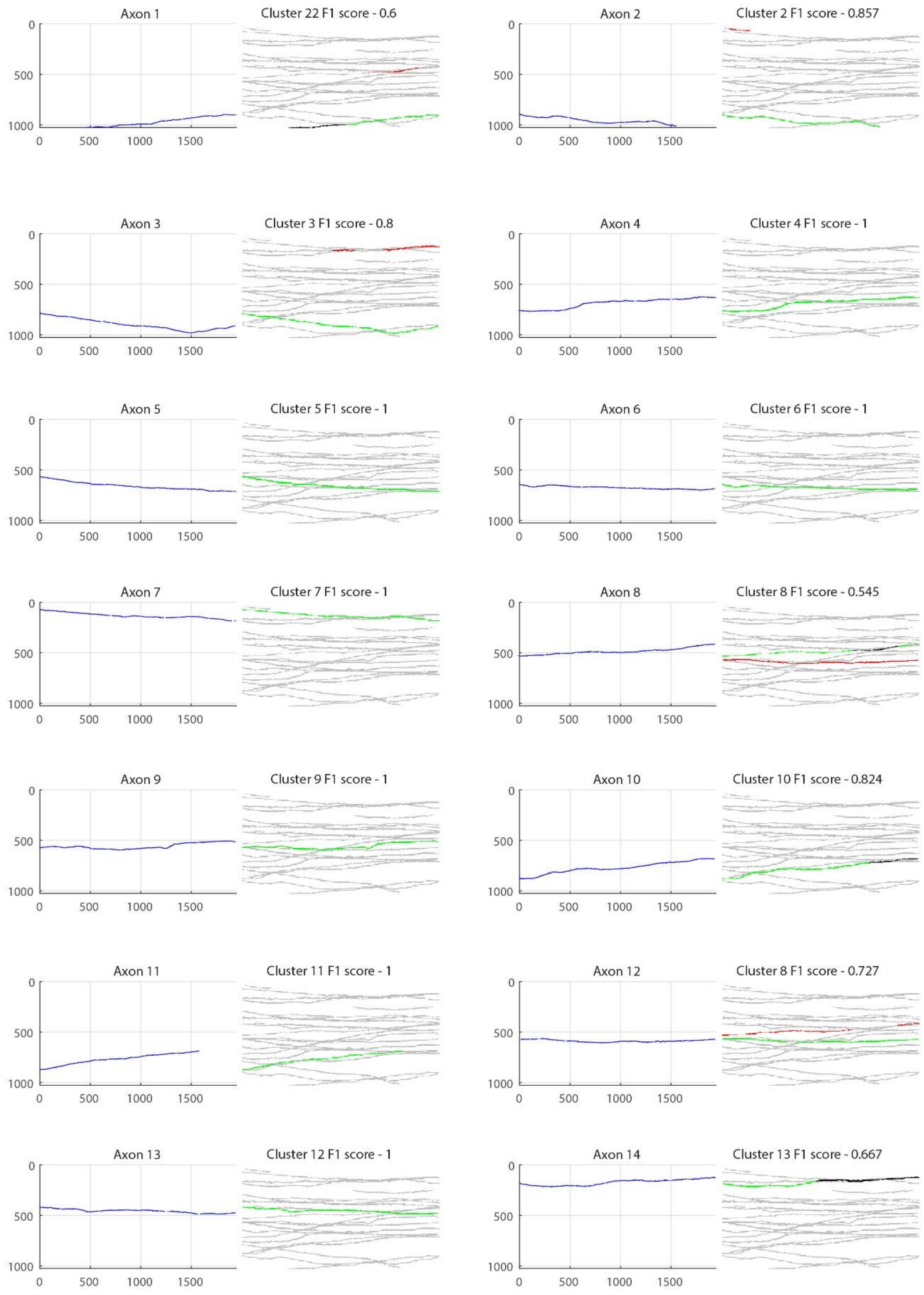

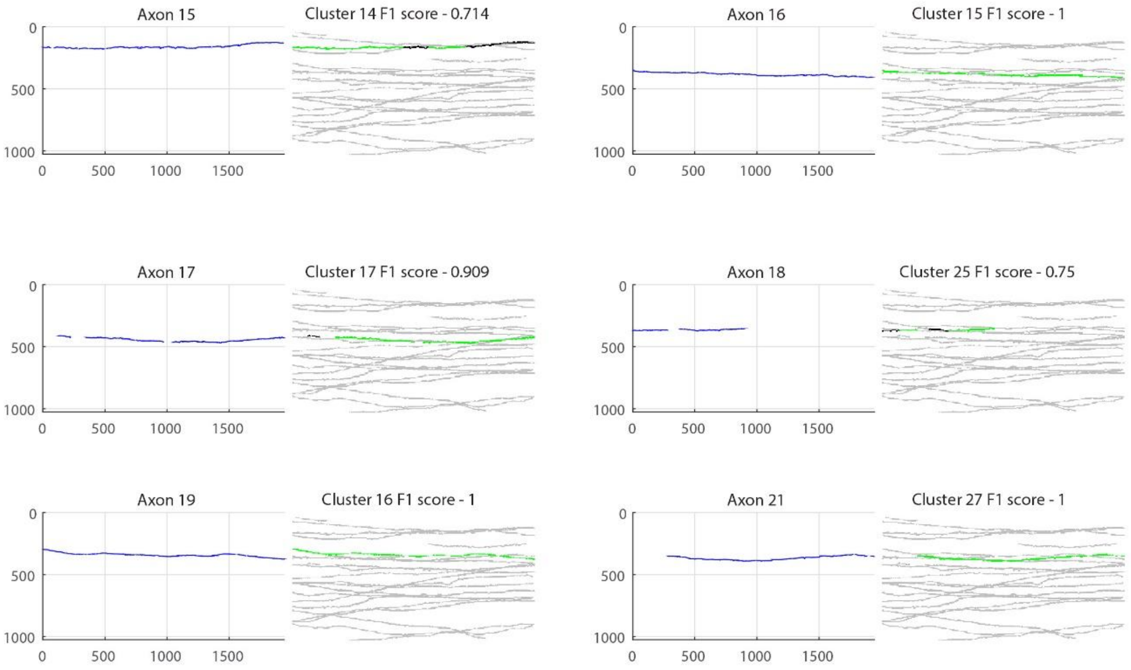

Axon 15
Cluster 14 F1 score - 0.714
Axon 16
Cluster 15 F1 score - 1
Axon 17
Cluster 17 F1 score - 0.909
Axon 18
Cluster 25 F1 score - 0.75
Axon 19
Cluster 16 F1 score - 1
Axon 21
Cluster 27 F1 score - 1
0
500
1000
1500

Supplementary Data 5 | Parameters used for linear unmixing in this study.
Parameters were determined based on the reference samples expressing only one type of XFP. Parameters were determined for each of the imaging conditions (objective lens, etc.).

Fig.1c,2e,6a, Supplementary Fig. 2c,d

	mTagBFP2	mTurquoise2	mAmetrine1.1	mNeonGreen	YPet	mRuby3	tdKatushka2
Ch1	1.00000000	0.14742014	0.00732682	0	0	0	0
Ch2	0.91251490	1.00000000	0.02033074	0	0	0	0
Ch3	0.23378902	0.59312861	1.00000000	0	0	0	0
Ch4	0	0	0	1.00000000	0.79405029	0	0
Ch5	0	0	0	0.41073063	1.00000000	0	0
Ch6	0	0	0	0	0	1.00000000	0.12450495
Ch7	0	0	0	0	0	1.23917092	1.00000000

Fig.1d,5e,8b, Supplementary Fig. 10a

	mTagBFP2	mTurquoise2	mAmetrine1.1	mNeonGreen	YPet	mRuby3	tdKatushka2
Ch1	1.00000000	0.15027173	0.00745169	0	0	0	0
Ch2	0.98396965	1.00000000	0.02032074	0	0	0	0
Ch3	0.26867463	0.60849324	1.00000000	0	0	0	0
Ch4	0	0	0	1.00000000	0.90969660	0	0
Ch5	0	0	0	0.41661105	1.00000000	0	0
Ch6	0	0	0	0	0	1.00000000	0.14882092
Ch7	0	0	0	0	0	0.96736655	1.00000000

Fig.7a,9a

	mTagBFP2	mTurquoise2	mAmetrine1.1	mNeonGreen	YPet	mRuby3	tdKatushka2
Ch1	1.00000000	0.10053646	0.01746663	0	0	0	0
Ch2	0.74372207	1.00000000	0.04297200	0	0	0	0
Ch3	0.10114225	0.29728307	1.00000000	0	0	0	0
Ch4	0	0	0	1.00000000	0.98084559	0	0
Ch5	0	0	0	0.32077865	1.00000000	0	0
Ch6	0	0	0	0	0	1.00000000	0.23110124
Ch7	0	0	0	0	0	0.66037713	1.00000000

Supplementary Fig.3

	mNeonGreen	YPet
U-MNIBA3	1.00000000	0.600969565
U-MYFPHQ	1.391649759	1.00000000

Supplementary Fig. 4

	mTagBFP2	mTurquoise2	mAmetrine1.1	mNeonGreen	YPet	mRuby3	tdKatushka2
Ch1	1.00000000	0.23399862	0.009498448	0	0	0	0
Ch2	0.904934547	1.00000000	0.02479776	0	0	0	0
Ch3	0.2292997	0.60211386	1.00000000	0	0	0	0
Ch4	0	0	0	1.00000000	0.810761000	0	0
Ch5	0	0	0	0.636619000	1.00000000	0	0
Ch6	0	0	0	0	0	1.00000000	0.385063416
Ch7	0	0	0	0	0	0.73555621	1.00000000
