# Supplementary material for: Automated neuronal reconstruction with super-multicolour Tetbow labelling and threshold-based clustering of colour hues
Source: Nat Commun. 2024 Jun 25;15:5279. doi: 10.1038/s41467-024-49455-y (PMC11199630; doi:10.1038/s41467-024-49455-y)
Supplement: Supplementary file 2 — Description of Additional Supplementary Files [file 41467_2024_49455_MOESM2_ESM.pdf]

Supplementary Movie 1

Visual representation of the dCrawler algorithm, related to Fig. 4.
