# Supplementary material for: Automated neuronal reconstruction with super-multicolour Tetbow labelling and threshold-based clustering of colour hues
Source: Nat Commun. 2024 Jun 25;15:5279. doi: 10.1038/s41467-024-49455-y (PMC11199630; doi:10.1038/s41467-024-49455-y)
Supplement: Supplementary file 3 — Reporting Summary [file 41467_2024_49455_MOESM3_ESM.pdf]

nature portfolio

Corresponding author(s): Takeshi Imai

Last updated by author(s): May 21, 2024

Reporting Summary

Nature Portfolio wishes to improve the reproducibility of the work that we publish. This form provides structure for consistency and transparency in reporting. For further information on Nature Portfolio policies, see our Editorial Policies and the Editorial Policy Checklist.

Statistics

For all statistical analyses, confirm that the following items are present in the figure legend, table legend, main text, or Methods section.

n/a	Confirmed	
☐	☒	The exact sample size (*n*) for each experimental group/condition, given as a discrete number and unit of measurement
☐	☒	A statement on whether measurements were taken from distinct samples or whether the same sample was measured repeatedly
☐	☒	The statistical test(s) used AND whether they are one- or two-sided<br>*Only common tests should be described solely by name; describe more complex techniques in the Methods section.*
☒	☐	A description of all covariates tested
☐	☒	A description of any assumptions or corrections, such as tests of normality and adjustment for multiple comparisons
☐	☒	A full description of the statistical parameters including central tendency (e.g. means) or other basic estimates (e.g. regression coefficient) AND variation (e.g. standard deviation) or associated estimates of uncertainty (e.g. confidence intervals)
☐	☒	For null hypothesis testing, the test statistic (e.g. *F*, *t*, *r*) with confidence intervals, effect sizes, degrees of freedom and *P* value noted<br>*Give P values as exact values whenever suitable.*
☒	☐	For Bayesian analysis, information on the choice of priors and Markov chain Monte Carlo settings
☒	☐	For hierarchical and complex designs, identification of the appropriate level for tests and full reporting of outcomes
☒	☐	Estimates of effect sizes (e.g. Cohen's *d*, Pearson's *r*), indicating how they were calculated

Our web collection on statistics for biologists contains articles on many of the points above.

Software and code

Policy information about availability of computer code

Data collection	All images were obtained using the Leica LAS X.
Data analysis	All codes are written using MATLAB 2021b (v9.11), and are publicly available in the GitHub repository https://github.com/mleiwe/QDyeFinder

For manuscripts utilizing custom algorithms or software that are central to the research but not yet described in published literature, software must be made available to editors and reviewers. We strongly encourage code deposition in a community repository (e.g. GitHub). See the Nature Portfolio guidelines for submitting code & software for further information.

Data

Policy information about availability of data

All manuscripts must include a data availability statement. This statement should provide the following information, where applicable:

- Accession codes, unique identifiers, or web links for publicly available datasets
- A description of any restrictions on data availability
- For clinical datasets or third party data, please ensure that the statement adheres to our policy

All of the image data acquired in this study has been deposited to SSBD:repository (https://ssbd.riken.jp/repository/346/, DOI: 10.24631/ssbd.repos.2024.05.346).
Neurolucida traces are available at Neuromorpho.org (https://neuromorpho.org/index.jsp): Data are associated with “Imai” archive and Pubmed ID of this paper.
Source data are provided with this paper.
Custom written codes are available at GitHub- https://github.com/mleiwe/QDyeFinder (Details are described in the "Data Availability" section in the manuscript).

Research involving human participants, their data, or biological material

Policy information about studies with human participants or human data. See also policy information about sex, gender (identity/presentation), and sexual orientation and race, ethnicity and racism.

Reporting on sex and gender	n/a
Reporting on race, ethnicity, or other socially relevant groupings	n/a
Population characteristics	n/a
Recruitment	n/a
Ethics oversight	n/a

Note that full information on the approval of the study protocol must also be provided in the manuscript.

Field-specific reporting

Please select the one below that is the best fit for your research. If you are not sure, read the appropriate sections before making your selection.

☒ Life sciences ☐ Behavioural & social sciences ☐ Ecological, evolutionary & environmental sciences

For a reference copy of the document with all sections, see nature.com/documents/nr-reporting-summary-flat.pdf

Life sciences study design

All studies must disclose on these points even when the disclosure is negative.

Sample size	Sample size was not pre-determined. We analyzed at least 3 animals each to confirm the reproducibility.
Data exclusions	High-quality images were used in the analysis. As noted in the paper, image quality is important for proper analysis. Criteria for exclusion in the analysis pipelines are detailed in the paper.
Replication	We have not compared biologically distinct sample groups and there was no need to show statistical significance.
Randomization	We have not compared biologically distinct sample groups
Blinding	We have not perform any blind analysis.

Reporting for specific materials, systems and methods

We require information from authors about some types of materials, experimental systems and methods used in many studies. Here, indicate whether each material, system or method listed is relevant to your study. If you are not sure if a list item applies to your research, read the appropriate section before selecting a response.

Materials & experimental systems

n/a	Involved in the study	
☒	☐	Antibodies
☐	☒	Eukaryotic cell lines
☒	☐	Palaeontology and archaeology
☐	☒	Animals and other organisms
☒	☐	Clinical data
☒	☐	Dual use research of concern
☒	☐	Plants

Methods

n/a	Involved in the study	
☒	☐	ChIP-seq
☒	☐	Flow cytometry
☒	☐	MRI-based neuroimaging

Eukaryotic cell lines

Policy information about cell lines and Sex and Gender in Research

Cell line source(s)	HEK293T
Authentication	The cell line was not authenticated.
Mycoplasma contamination	The cell line was not tested for mycoplasma contamination.
Commonly misidentified lines (See ICLAC register)	n/a

Animals and other research organisms

Policy information about studies involving animals; ARRIVE guidelines recommended for reporting animal research, and Sex and Gender in Research

Laboratory animals	ICR mice (4 week-old, male and female),<br>C57BL/6N (7-13 week-old, male and female)<br>Pcdh21-Cre (8-13 week-old, male and female)
Wild animals	n/a
Reporting on sex	No sex-based analysis was performed. We did not need to consider sex differences as we used mice just for obtaining sets of fluorescent labelled neurons. Sex is reported for all the analyses (except for young mice).
Field-collected samples	n/a
Ethics oversight	All animal experiments were reviewed and approved by the Institutional Animal Care and Use Committee of Kyushu University

Note that full information on the approval of the study protocol must also be provided in the manuscript.

Plants

Seed stocks	n/a
Novel plant genotypes	n/a
Authentication	n/a
